# Hepatitis A Virus Cellular Receptor 1 (HAVcr-1) Initiates Prostate Cancer Progression in Human Cells via Hepatocyte Growth Factor (HGF)-Induced Changes in Junctional Integrity

**DOI:** 10.3390/biom12020338

**Published:** 2022-02-21

**Authors:** Emily A. Telford, Andrew J. Sanders, Sioned Owen, Fiona Ruge, Gregory M. Harrison, Wen G. Jiang, Tracey A. Martin

**Affiliations:** Cardiff China Medical Research Collaborative, Division of Cancer and Genetics, Cardiff University School of Medicine, Cardiff University, Cardiff CF14 4XN, UK; telfordej@cardiff.ac.uk (E.A.T.); sandersaj1@cardiff.ac.uk (A.J.S.); sioned.owen@southwales.ac.uk (S.O.); ruge@cardiff.ac.uk (F.R.); cardiffchina@cardiff.ac.uk (G.M.H.); jiangw@cardiff.ac.uk (W.G.J.)

**Keywords:** HAVcR-1, prostate cancer, metastasis, migration, junctions, prognosis

## Abstract

Background: HAVcR-1 has been linked to cancer aetiology and may regulate junctional complexes, with its role in prostate cancer still unexplored. This study aims to investigate the expression of HAVcR-1 in prostate cancer samples and the exploration of the cellular/molecular impact of HAVcR-1. Methods: Levels of HAVcR-1 ectodomain in the serum of prostate cancer patients were compared to healthy controls, and assessed as the total protein and gene expression of HAVcR-1 and tissues sections. The manipulation of HAVcR-1 levels within prostate cancer cell lines determined changes in cell behaviour using in vitro cell models and barrier function assays. Protein/phosphoprotein levels were assessed using Western blotting. Results: Levels of HAVcR-1 ectodomain from serum were decreased in patients with prostate cancer. Ectodomain levels correlated with the Gleason score. Histologically, the total protein/gene expression of HAVcR-1 was overexpressed in prostate cancer. The overexpression of HAVcR-1 in prostate cancer cell lines resulted in key changes in cell behaviour and the phosphorylation of β-catenin with a concurrent decrease in membranous E-cadherin, increased nuclear β-catenin and increased cyclin D1 protein expression, which were associated with HGF-promoted changes in the barrier function. Conclusions: HAVcR-1 expression and ectodomain release coincides with the presence of prostate cancer; thus, indicating HAVcR-1 as a potential biomarker to aid in diagnostics, and implicating HAVcR-1 in the dysregulation of junctional complexes.

## 1. Introduction

Prostate cancer is the second most common cancer in males worldwide and the most common cancer in males in the UK, with approximately 1,111,000 and 43,436 new cases per year, respectively [1,2]. Diagnostic techniques, however, are still reliant on the inherently flawed PSA blood test, which has limited associated risks in comparison to other invasive testing, such as prostate biopsies, which may result in a subsequent infection and urinary incontinence [3,4]. It is, therefore, important to identify novel biomarkers that can be used to improve the accuracy of low invasive testing. Of greater significance is the current inability to differentiate between low-risk progression and high-risk progression prostate cancer at an early curable stage [5], whereby low-risk progression prostate cancers are those that are unlikely to grow or metastasize outside of the prostate for many years and, therefore, have a limited risk of morbidity or mortality, whilst high-risk progression prostate cancers are those that are likely to grow and progress to metastatic disease, resulting in an increased morbidity and mortality [6]. The problem with not being able to identify high-risk progression prostate cancer is that it results in the overtreatment of low-risk progression prostate cancer and the unnecessary associated morbidity [5]. This highlights the necessity of identifying biomarkers to categorize tumours that are likely to progress at an early stage to ensure treatment is provided. 

The hepatitis A virus cellular receptor (HAVcR-1) is the cellular receptor for the hepatitis A virus and hepatotropic picornavirus, the cause of acute hepatitis A in humans [7]. HAVcR-1 is also termed T-cell immunoglobulin and a mucin domain containing molecule 1 (TIM-1) and kidney injury molecule-1 (KIM-1). HAVcR-1 is expressed on every tested human organ, including the liver, small intestine, colon and spleen, as well as high expression in the kidney and testis, however, the natural function of HAVcR-1 has not been fully investigated [7]. 

HAVcR-1 has been found to be upregulated in certain cancers, including breast, ovarian, colon and renal cancer [8,9,10]. HAVcR-1 is, therefore, a molecule of interest for cancer diagnosis and a potential target for cancer therapies. HAVcR-1 is proteolytically cleaved proximal to the cell membrane to release an ectodomain [11,12]. This HAVcR-1 ectodomain can be secreted into urine from certain tissue types, and this release is increased in renal cell carcinoma [13]. The HAVcR-1 ectodomain is, therefore, a potential biomarker for certain cancers. HAVcR-1 expression and ectodomain release in cancer is still poorly categorized. There is little known about its usefulness as a biomarker for prostate cancer diagnosis, progression and prognosis. Furthermore, there is a lack of study into the release of the HAVcR-1 ectodomain into the circulation and the use of this as a potential biomarker for use in blood tests for cancer diagnosis and monitoring.

HAVcR-1 has been linked to tight junctions (TJs), which have an important role in the prevention of cancer metastasis. Evidence currently suggests that HAVcR-1 overexpression seen in cancer is linked to TJ disruption and, therefore, links HAVcR-1 to cancer metastasis [9]. A number of virus receptors have been found to be associated with junctional structures, including TJs and adherens junctions (AJs), and investigations into the association of HAVcR-1 with junctional structures found via immunoprecipitation that the 50 kDa HAVcR-1 associates with the C terminal of Zonula Occludens-1 (ZO-1) and, to a lesser extent, Zonula Occludens-2 (ZO-2), as well as the N-terminal of occludin and RhoC (Ras homolog gene family, member C, Rho GTPase) [14]. Due to the importance of these molecules in TJs, it is possible that HAVcR-1 is also involved in the TJ complex in endothelial and epithelial cells. The overexpression and knockdown analysis of HAVcR-1 in a human umbilical cord cell line (HECV cells) suggests the importance of HAVcR-1 expression in the hepatocyte growth factor (HGF)-mediated breakdown of TJ, as shown by the decreased trans-endothelial resistance (TER) in HAVcR-1 overexpressed HECV cells in comparison to HAVcR-1 knockdown HECV endothelial cells when treated with HGF [14]. The dual immunofluorescence of HAVcR-1 and ZO-1 showed an increased expression and concentrated disruption of ZO-1 in cell–cell junctions in knockdown HECV cells in comparison to wild-type HECV cells when treated with HGF. Therefore, it appears likely that both HGF and HAVcR-1 act on the same pathway responsible for the integrity and maintenance of TJs [14]. The overexpression of HAVcR-1 in cell lines results in decreased TJs, and HAVcR-1 overexpression in cancer is likely to also result in decreased TJs, which may mediate metastasis.

The role of HAVcR-1 in cancer development and progression is an active area of research; however, the role of HAVcR-1 in prostate cancer has not been fully investigated. Therefore, in this study, we sought to determine whether HAVcR-1 and/or the HAVcR-1 ectodomain would provide an effective biomarker for prostate cancer diagnosis and that through the dysregulation of epithelial cell adhesion, HAVcR-1 would contribute to the development and progression of the metastatic disease of prostate cancer. Furthermore, this work focuses on evaluating whether there is a potential to use HAVcR-1 as a prostate cancer biomarker and/or a viable therapeutic option to prevent/treat prostate cancer metastasis and to establish the role of HAVcR-1 in cellular adhesion complexes, cellular behaviour and the effects of HGF in conjunction with HAVcR-1 expression on cellular adhesion complexes and cellular behaviour.

## 2. Materials and Methods

### 2.1. Cell Lines

This study used PZ-HPV-7 (an immortalized prostate epithelial cell line), CA-HPV-10 (an immortalized prostate adenocarcinoma cell line) and LNCaP, PC-3 and Du145 (metastatic prostate cancer cell lines). In addition, the HECV vascular endothelial cell line was used. All cell lines were purchased from the American Tissue Culture Collection (ATCC) (Manassas, VA, USA) at the commencement of this study. PC-3, Du145 and HECV cell lines were maintained in DMEM medium (Sigma-Aldrich, Gillingham, Dorset, UK). LNCaP clone FGC cell line was maintained in RPMI-1640 medium (Sigma-Aldrich, Gillingham, Dorset, UK). PZ-HPV-7 and CA-HPV-10 cell lines were maintained in Keratinocyte-SFM (Sigma-Aldrich, Gillingham, Dorset, UK).

### 2.2. Polymerase Chain Reaction (PCR) Primers

Primers used were designed using Primer-BLAST available from NCBI. Reverse primers used for quantitative polymerase chain reaction (qPCR) included the addition of a z-sequence on the 5′ end of the primer (Amplifuor™ Uniprimer™) consisting of a 3′ complimentary sequence that pairs with the z-sequence (ACTGAACCTGACCGTACA) present in qPCR reverse primers, as well as a 5′ hairpin loop labelled with a fluorophore reporter (FAM). Custom-designed primers were synthesized by Sigma-Aldrich (Gillingham, Dorset, UK), diluted to 100 µm in PCR H_2_O and stored at −20 °C. Forward and reverse primers and forward primers for qPCR were further diluted 1:10 before use. Reverse qPCR primers were further diluted 1:100 before and all diluted primers were temporarily stored at 4 °C. Full sequences were as follows: Target Gene Forward (F) and Reverse (R) Primer Sequences 5′-3′ Cycle Number was 30. HAVcR1 F: CAACAACAAGTGTTCCAGTG 436 bp; R: GCATTTTGCAAAGCTTTAAT. GAPDH F: GGCTGTTTTAACTCTGGTA 475 bp; R: GACTGTGGTCATGAGTCCTT. ZO-1 F: CCACATACAGATACGAGTCCTC 533 bp; R: TGGCTTATGCTGAGATGAAGG. ZO-2 F: CTGACATGGAGGAGCTGA; R: GAGACCATACTCTTCGTTCG. CLDN1 F: ATGGCCAACGCGGGGC 636 bp; R: TCACACGTAGTCTTTCC. CLDN2 F: TATAGCACCCTTCTGGGCCT 432 bp; R: CCTTGGAGAGCTCCTTGTGG. CLDN3 F: ATGCAGTGAAGGTGTACGA; R: TGGTGGCCGTGTACTTCTTC. CLDN4 F: TGGGAGGGCCTCTGGATGAA 422 bp; R: TGGTGGCCGTGTACTTCTTC. CLDN7 F: ATAACCCTTTGATCCCTACC 113 bp; R: ACTGAACCTGACCGTACAACAGG. CLDN9 F: CTTCATCGGCAACAGCATCG 339 bp; R: AAGTCCTGGATGATGGCGTG. JAM1 F: AACAAGATCACAGCTTCCTA 600 bp; R: CTTACTCGAAGTCCCTTTCT. OCLN F: ATGTCATCCAGGCCTC 579 bp; R: ATAGACAATTGTGGCA. α-catenin F: CACAGAGAAGGTTCTGGAAG 518 bp; R: CCGATGTATTTTTGAGTGGT. β-catenin F: AAAGGCTACTGTTGGATTGA 649 bp; R: TCCACCAGAGTGAAAAGAAC. Cyclin D1 F: CGGTGTCCTACTTCAAATGT 721 bp; R: ACCTCCTCCTCCTCCTCT. EPLIN F: TCAAACTAAGATTCTCCGGG 30 875; R: TCGGGGCATCTTCTACC. GSK3β F: ATGTTTCGTATATCTGTT 534 bp; R: GGTGGAGTTGGAAGCTGATG. Primer sequences used in qPCR, reverse primer z-sequences are highlighted in bold. Target Gene Forward (F) and Reverse (R) Primer Sequences 5′-3′. HAVcR-1 F: GACAATGTTTCAACGA 99 bp; R: ACTGAACCTGACCGTACATGGAGGAACAAA. GAPDH F: CTGAGTACGTCGTGGAGTC 93 bp; R: ACTGAACCTGACCGTACACAGAGATGATGACCCTTTTG.

### 2.3. Antibodies

Primary antibodies were purchased and used at a dilution of 1:500 (unless indicated otherwise) as follows: HAVcR-1 Pab13202, (Abnova, Heyford, Oxon, UK); TIM-1/HAVcR-1 AF1817, E-cadherin 17029 (R&D Systems, Abingdon, Oxon, UK); TIM-1 (N-13 HAVcR-1) SC47495, GAPDH SC32233, Claudin-1 SC17658, Claudin-7 SC177670, Occludin SC8145, ZO-1 SC8146, PKM2 SC65176, cyclin D1 SC753 (Insight Biotechnology LTD, Middlesex, UK); α-catenin C1620 (BD Transduction Laboratories, San Jose, CA, USA); β-catenin 8415 (Sigma-Aldrich, Gillingham, Dorset, UK); Eplin A300-103A (Bethyl Labs, Montgomery, TX, USA). Secondary antibodies were prepared as per manufacturer’s instructions and were purchased as follows: Anti-Mouse IgG A4416, anti-Rabbit IgG A6154, anti-Goat IgG A5420 (Sigma-Aldrich, Gillingham, Dorset, UK); Biotinylated anti-Mouse IgG (Vector Labs, Orton Southgate, Peterborough, UK); AlexaFluor 488 anti-Rabbit, anti-Mouse, anti-Goat and AlexaFluor 594 anti-Rabbit, anti-Goat, DAPI (Thermo Fisher Scientific, Cramlington, UK).

### 2.4. Human Prostate Cancer Serum Samples

Prostate cancer serum samples (*n* = 237) and breast cancer serum samples (*n* = 148) were obtained from Wales Cancer Bank (WCB). Whole blood (*n* = 9) was obtained from the Welsh Blood Service or obtained from male volunteers with informed consent (*n* = 4). Serum from volunteers was extracted via centrifugation at 15,000× *g* for 10 min and stored at −80 °C.

### 2.5. Patient Tissue Samples

Prostate cancer samples (*n* = 2) and normal control samples (*n* = 2) were collected at the University Hospital of Wales between January 2003 and 2006. Sections were collected with informed patient consent and with ethical approval from the South East Wales Research Ethics Committee (Panel C) under the project title “Hepatocyte growth factor (HGF) and its regulators on the behavior of invasive/metastatic prostate cancer”. Ethics no: 03/5048.

### 2.6. HAVcR-1 Knockdown/Overexpression

Knockdown of HAVcR-1 was achieved using ribozyme technology. The HAVcR-1 specific ribozyme insert was cloned into the plasmid using the pEF6/V5-His TOPO TA expression kit (Invitrogen, Paisley, Scotland, UK). Ribozymes and the expression cassette were constructed as described in work by Martin et al. (2011) [14].

### 2.7. RNA Extraction and PCR

Cells were lysed and RNA extracted using EZ-RNA kit (Geneflow, Staffordshire, UK). RNA was reverse transcribed to cDNA using the GoScript™ Reverse Transcription System (Promega, Southampton, UK). PCR was carried out using GoTaq Green Master Mix (Promega, Southampton, UK) with specific primers detailed above using a thermocycler geneAmp PCR system 2700 (Thermo Fisher Scientific, Cramlington, UK). All genes were normalized to the GAPDH housekeeping gene; thus, for every cDNA sample, a PCR reaction with primers specific for GAPDH was carried out. Furthermore, for every primer set, a negative control PCR reaction was carried out, whereby the PCR mix contained no cDNA. Agarose gel electrophoresis was used to separate DNA fragments according to size. Samples were loaded onto a 2% (*w*/*v*) agarose gel (Sigma-Aldrich, Gillingham, Dorset, UK). Precision FAST 2X qPCR Master Mix with ROX (Primer Design, Southampton, UK) and Amplifuor™ Uniprimer™ Universal System (Intergen Company^®^, Bronx, NY, USA) were used to carry out qPCR. qPCR was carried out for each sample using primers specific to the housekeeping gene GAPDH, which was then used for normalizing. The qPCR mixes were compiled in triplicate in a microamp^®^ Fast Optical 96-well reaction plate with barcode (Applied Biosystems, Carlsbad, CA, USA) and covered with MicroAmp^®^ Optical Adhesive film (Thermo Fisher Scientific, Cramlington, UK). qPCR was carried out using the StepOne Plus Real-Time PCR System (Thermo Fisher Scientific, Cramlington, UK). The cycle at which the fluorescent signal reached a particular threshold, known as the CT value, was then given and this was then analysed using ∆∆ CT normalized to the GAPDH housekeeping gene.

### 2.8. Protein Detection and Analysis

Cell media were aspirated, and cells were washed with PBS before being lysed with lysis buffer. The amount of lysis buffer used depended on culture size. Cell lysates were then incubated on ice for 5 min and transferred to microfuge tubes. Cell lysates were then rotated for 30 min on a Labinoco LD79 Test-tube Rotator (Wolf Laboratories, York, UK) prior to centrifugation at 12,000× *g* for 15 min at 4 °C. Supernatant (protein lysate) was then transferred into a fresh microfuge tube and either stored at −20 °C ready for protein sample quantification or equal volumes of LaemmLi 2 X Concentrate (Sigma-Aldrich, Gillingham, Dorset, UK) added prior to boiling at 100 °C for 10 min. The Bio-Rad DC™ Protein Assay Kit (Bio-Rad, Hertfordshire, UK) was used for protein sample quantification. SDS-PAGE was undertaken on an acrylamide gel composed of a 10% (*v/v*) running gel and 5% (*v/v*) stacking gel in an OmniPAGE VS10DYS Vertical Electrophoresis System (OmniPAGE, Cleaver Scientific Ltd., Rugby, UK). Samples were then transferred from the acrylamide gel to a PVDF Transfer Membrane (Merck Millipore, Sigma-Aldrich, Gillingham, Dorset, UK) using the Mini Trans-Blot^®^ Cell (Bio-Rad, Hertfordshire, UK) wet transfer system. Standard Western blotting was carried out. EZ-ECL Chemiluminescent Detection Kit (Geneflow, Staffordshire, UK) was used for protein visualization.

### 2.9. Kinexus™ Antibody Microarray Protein Preparation

In preparation for a Kinexus™ Antibody Microarray, PZ-HPV-7 pEF6 and PZ-HPV-7 HAVcR1EXP cells were cultured in 10 cm dishes. When confluent cells were washed twice in PBS, 100 μL Kinexus™ Antibody array lysis buffer was added to lyse cells, followed by quantification using fluorescamine reagent (F9015, Sigma-Aldrich, Gillingham, Dorset, UK). Protein samples were then diluted to 4 mg/mL using Kinexus™ Antibody array lysis buffer to a final volume of 300 μL. Samples were then stored at −20 °C prior to being shipped to Kinexus Bioinformatics, Vancouver, Canada, for the Kinexus™ Antibody Microarray. The Kinexus™ KAM880 Protein Array service provided by Kinexus Bioinformatics Ltd. (Vancouver, BC, Canada) was utilized for this project. The Kinexus™ KAM880 Protein Array uses microarray chips which contain two sets of 877 antibodies. The percentage change from control (%CFC) was calculated, whereby treated referred to PZHPV-7 HAVcR-1EXP and control referred to PZ-HPV-7 pEF6. Percentage error, Z-scores and Z-ratios were also calculated. Significance was based on z-values of ≤−1.65 or ≥1.65.

### 2.10. Visualization of Proteins Using Immunofluorescence

Cells were seeded into 8-well glass Millicell EZ slides (Merck Millipore, Sigma-Aldrich, Gillingham, Dorset, UK) at 5 X 10^4^ cells per well in 500 μL medium. Slides were then incubated at 37 °C in a 95% (*v/v*) humidified atmosphere of 5% (*v/v*) CO_2_ until cells were confluent. Once confluent, culture medium was removed and cells were washed with PBS and fixed in 500 μL 100% ice-cold ethanol per well and left at −20 °C. Ethanol was then removed and cells were wash 3 times with PBS for 5 min per wash and permeabilized by adding 500 μL 0.1% Triton X-100 (Sigma-Aldrich, Gillingham, Dorset, UK) per well for 1–5 min, depending on protein of interest, at room temperature. Cells were then washed 3 times in PBS for 5 min per wash and blocked using blocking buffer, consisting of 7.5% (*v/v*) donkey serum (D9663, Sigma-Aldrich, Gillingham, Dorset, UK) in PBS, at 300 μL per well for 6 h at room temperature. Primary and secondary antibodies were used according to manufacturer’s instructions, slides were mounted with FluorSave™ (345789, Sigma-Aldrich, Gillingham, Dorset, UK) and a cover slip. Slides were then left to set in the dark at 4 °C overnight and visualized/imaged using the Hamamatsu Orca ER digital camera and the Olympus BXSA microscope at 100× magnification. Merged images were then created using Adobe Photoshop software.

### 2.11. Immunohistochemical Staining (IHC) of Patient Tissues

Cryosections were stored at −80 °C. These were allowed to thaw at room temp for approximately 15 min prior to being fixed with dried acetone (10162180, Fisher Scientific, Loughborough, UK) for 15 min, air dried for 15 min and washed 3 times with PBS for 5 min per wash. Cryosections were then incubated with blocking diluent (0.1% (*v/v*) BSA, 0.01% (*v/v*) Marvel, 10% (*v/v*) horse serum and 90% (*v/v*) PBS) for 1 h in a humidified box at room temperature. Sections were then incubated in a humidified chamber for 1 h in primary antibody diluted in blocking diluent to a final concentration of 2 µg/mL or blocking diluent for negative controls. Sections were again washed 3 times in PBS for 5 min per wash and then incubated for 30 min in ABC biotinylated secondary antibody diluted in blocking diluent in a humidified chamber for 30 min. Sections were washed 3 times in PBS for 5 min per wash, incubated in a humidified chamber for 30 min in ABC reagent provided in the Vectastain Universal Elite ABC kit (Vector, Peterborough, UK), washed 3 times in PBS and developed with diaminobenzidine substrate (DAB) (Abcam, Cambridge, UK) (90% (*v/v*) 10% (*v/v*) DAB and 6 μL hydrogen peroxide for 10 min). Sections were then washed in H_2_O, counterstained in Ehrlich’s haematoxylin for 5–10 min and washed in H_2_O. Visualization and imaging of sections was performed using the Leica DM10000LED microscope with a MC120 HD camera and Leica Application Suite (version 3.0.0) software (Leica Microsystems, UK). Localization and intensity of staining were judged blindly by two people independent of one another.

### 2.12. Enzyme-Linked Immunosorbent Assay (ELISA)

ELISA was performed using Human TIM-1 (HAVCR1) ELISA Kit (Thermo Fisher Scientific, Cramlington, UK). Serum samples were diluted 1:2 in Diluent B and 100 μL of each sample and provided standards were placed into appropriate wells of the provided 96-well plate. The absorbance was measured on an ELx800 Absorbance Reader (BioTek, Swindon, UK) at 450 nm. The absorbance of standards was then used to form a standard curve and this was used to calculate the protein concentration of samples.

### 2.13. In Vitro Cell Function Assays to Ascertain Changes in Cell Behaviour after HAVcR-1 Knockdown

Growth assays—Cells were seeded in triplicate into 24-well plates at 1 X 103 cells per well in 1 mL of cell medium and incubated at 37 °C in a 95% (*v/v*) humidified atmosphere of 5% (*v/v*) CO for 1, 3 and 5 days. After incubation, cells were washed with PBS and then fixed, stained and imaged (crystal violet, V5265, Sigma-Aldrich, Gillingham, Dorset, UK). Cell growth was presented as the fold change in cell number from the 1 day time point. Adhesion assays—Conducted using Matrigel™ basement membrane matrix (BD Biosciences, Oxford, UK) diluted to 0.05 mg/mL in cell medium and 100 μL was loaded into each well of a 96-well plate. This was then dehydrated at 56 °C. Following rehydration with serum-free media, cells were seeded at 5000 cells per well in 200 μL of cell medium and incubated for 30 min. The cell medium was then discarded, the cells were washed with PBS and stained using crystal violet before counting or die removal using 10% (*v/v*) acetic acid, and absorbances were measured at 540 nm using the ELx800 Absorbance Reader (BioTek, Swindon, UK). Invasion potential—Assessed using a standard invasion model, with a 0.8 µm pore ThinCert™ 24-well plate inserts (Greiner Bio-One Ltd., Gloucestershire, UK). Matrigel™ basement membrane matrix (BD Biosciences, Oxford, UK) was diluted in serum-free medium to 0.5 mg/mL and 100 μL loaded into each insert to replicate the extracellular matrix. Cell seeding was at 3 X 10^4^ cells per insert in 500 μL of serum-free medium. Cells that had invaded the Matrigel™ layer and had migrated through to the underside of the ThinCert™ 24-well plate inserts were stained using crystal violet before counting. Changes in TJ function—Assessed using transepithelial resistance (TER) and paracellular permeability (PCP). TER used 0.4 µm pore ThinCert™ 24-well plate inserts (Greiner Bio-One Ltd., Gloucestershire, UK) in 24-well plates. Cells were seeded into inserts at 5 X 103 cells per insert in 500 μL of cell medium with 1.5 mL medium in the well outside of the insert. Cells were incubated until confluent. Resistance across the membrane was then measured in triplicate immediately afterwards using the EVOM² Epithelial Volt/Ohm Meter (World Precision Instruments, Hitchin, Hertfordshire, UK). Where treatments were applied, resistance was also measured every hour after the initial measurement for 10 h. Data were then converted to Ω·cm^2^ by the multiplication of measured resistance by the surface area of the ThinCert™ 24-well plate inserts. PCP used 0.4 µm pore ThinCert™ 24-well plate inserts (Greiner Bio-One Ltd., Gloucestershire, UK) in 24-well plates. Cells were seeded into inserts at 5 X 103 cells per insert. Medium was then replaced and 0.2 mg/mL of tetramethylrhodamine isothiocyanate (TRITC)–dextran conjugate with an average molecular weight of 40 kDa (42874, Sigma-Aldrich, Gillingham, Dorset, UK) and 0.2 mg/mL of fluorescein isothiocyanate (FITC)–dextran conjugate with an average molecular weight of 10 kDa (FD10S, Sigma-Aldrich, Gillingham, Dorset, UK) was added to each insert. Immediately after media change and every hour thereafter until 10 h, 20 μL of cell medium from outside of the inserts was transferred into a black 96-well cell culture microplate (Greiner Bio-One) in duplicate. Fluorescence was then measure using the GloMax^®^ Multi Detection System (Promega, Southampton, UK) at excitation 520 and emission 580–640 for TRITC–dextran and excitation 940 and emission 510–570 for FITC–dextran. Measurements were then normalized to the 0 h time point measurement via subtraction and a statistical analysis was performed. Standard wound healing/scratch model to assess migration—Briefly, cells were seeded in quadruplicate into 24-well plates at 5 X 103 cells per well, a manual scratch was performed and images were then taken at 5× magnification to give the 0 h time point. Images were then taken every hour after this up to 10 h. Images were then analysed using ImageJ software to give wound area, which was then used to calculate the percentage change in area from the 0 h time point (presented as percentage wound closure). Electric cell–substrate impedance sensing (ECIS) to assess changes in cell behaviour—ECIS was performed using 96W1E+ plates (ECIS Cultureware™, IBIDI, Martinsried, Germany) and the ECIS^®^ Z-theta model instrument (IBIDI, Martinsried, Germany). Wells were inoculated with 5 X 10^4^ cells per well in 300 μL recommended cell medium. The behaviour for cell monolayers was then electrically monitored at 7 predefined frequencies (1, 2, 4, 8, 16, 32 and 64 kHz). At 25 h, an electrical wound of 60,000 Hz and 3000 µA was applied to the cell monolayers for 30 s. The resulting changes to the cell monolayers were continuously monitored, at the frequencies stated previously, during wounding and for 17 h post wounding. The data collected were analysed as fold change from time 0 h for initial attachment and spreading and from time 25 h for wound healing for resistance and capacitance at 1 kHz and 64 kHz, respectively, as well as well as for Rb and alpha. Effect of HGF—Functional assays where HGF treatment used cells were either treated with 40 ng/mL HGF or an equal amount of 0.1% BSS in PBS as the control. Treatment was conducted at time 0 in all cases with this being at seeding for growth, invasion and adhesion, immediately after scratch formation with migration, at the same time as fluorescent dextran conjugates for PCP and immediately after base line (time 0 h) readings for TER.

### 2.14. Statistical Analyses

Microsoft Excel was used for statistical analysis of the majority of data utilizing a two-tailed unpaired Student’s t-test. For patient serum samples, Graphpad Prism (version 6, GraphPad Software Inc., San Diego, CA, USA) was used, whereby a D’Agostino–Pearson omnibus K2 normality test was performed on columns to assess normality. If data were of a normal distribution, a two-tailed unpaired Student’s *t*-test or *T*-test with Welsh correction was performed for the comparison of two datasets or a one-way ANOVA for the comparison of three or more datasets. If data were not of a normal distribution, a Mann–Whitney U test was performed for the comparison of two datasets or a Kruskal–Wallis test was performed for the comparison of three or more datasets. Linear regression analysis using Graphpad Prism 6 software was utilized to address significance between slopes. In all cases, *p* values of ≤0.05 were considered significant and represented on graphs by *. Where *p* ≤ 0.01 or *p* ≤ 0.001, the representation of ** or *** was used, respectively.

## 3. Results

### 3.1. Expression of HAVcR-1 in Human Serum and Tissues

**Figure 1:**
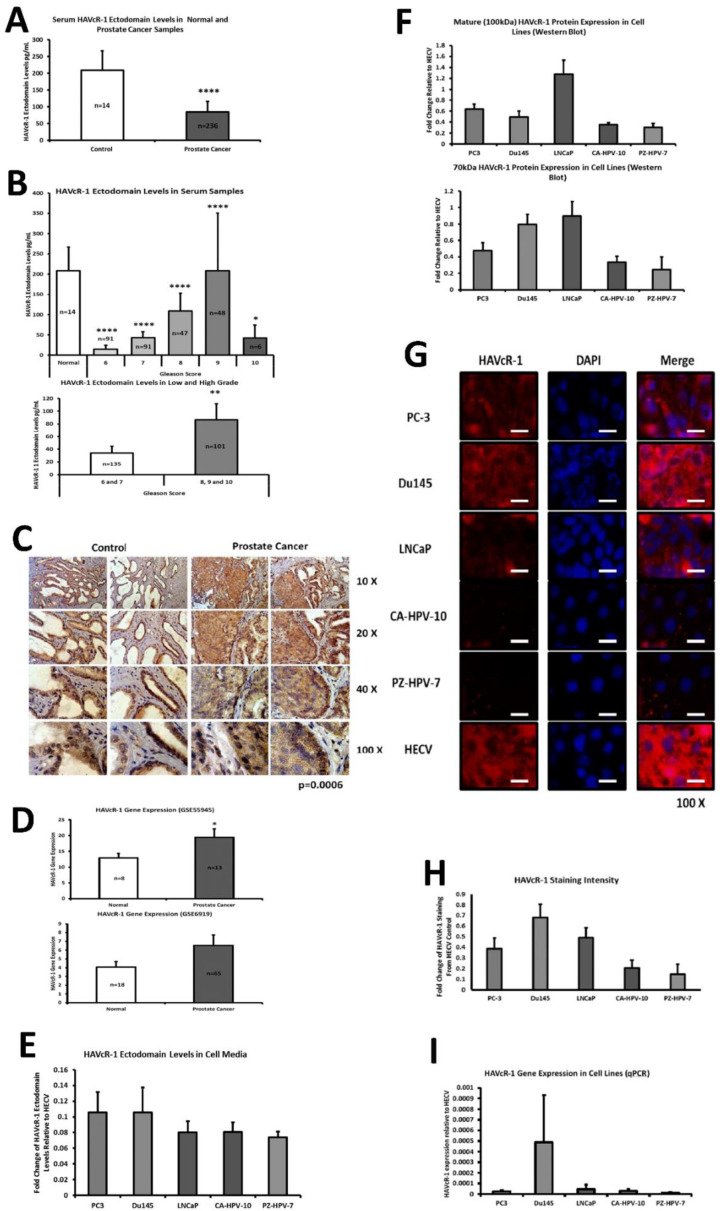
HAVcR-1 expression in human serum and tissues. (**A**) Serum HAVcR-1 Ectodomain analysed for HAVcR-1 ectodomain using Human TIM-1 (HAVCR1) ELISA Kit. Graph shows the difference in means of levels between healthy controls and prostate cancer patients. *p* ≤ 0.05 was considered significant. **** represents *p* ≤ 0.0001, error bars show SEM and *n* numbers are shown within bars. (**B**) Serum HAVcR-1 ectodomain levels ELISA: Top—results were analysed to assess differences in levels in serum samples between control group and Gleason score of 6, 7, 8, 9 and 10 prostate cancer; bottom—between low Gleason score (6 and 7) and high Gleason score (8, 9 and 10). *p* ≤ 0.05, *p* ≤ 0.01 and *p* ≤ 0.0001 are represented by *, ** and ****, respectively. Graphs show the means with error bars showing SEM and *n* numbers are shown within bars. (**C**) Tissue samples stained for HAVcR-1 protein expression using IHC and intensity of staining analysed. *p* ≤ 0.05 was considered significant and the *p* value stated next to images. (**D**) GEO datasets analysis of HAVcR-1 gene Expression in prostate cancer in GEO datasets A GSE55945 and B GSE6919. Data shown are the means with error bars showing SEM and *n* numbers are shown within bars. (**E**) HAVcR-1 ectodomain release from prostate cell lines to assess differences in HAVcR-1 ectodomain level between different cell lines and shown as fold change relative to HECV-positive control (not shown on graph). Graph shows the means of three independent experiments with error bars showing SEM. (**F**) HAVcR-1 protein expression in prostate cell lines, graphs show band intensity as quantified by ImageJ software for B the ~100 kDa mature protein and C the ~70 kDa immature protein. (**G**) HAVcR-1 protein staining in prostate cell lines, images were taken at 100× magnification. Scale bars are representative of 20 µm. A images are representative of three independent experiments and show fluorescence emission correlating to HAVcR-1 expression or nuclear staining and a merged image of both. (**H**) Graph shows quantitative analysis of immunofluorescent staining of HAVcR-1. (**I**) qPCR gene analysis of HAVcR-1 mRNA expression was normalised to GAPDH and is shown as fold change relative to HECV-positive control (not shown on graph).

Serum HAVcR-1 ectodomain levels in prostate cancer: To investigate the release of the HAVcR-1 ectodomain into the circulation during the occurrence of prostate cancer, HAVcR-1 ectodomain levels were assessed in serum samples from patients with prostate cancer and from healthy controls using ELISA. HAVcR-1 ectodomain levels were decreased in serum samples from the prostate cancer patient group (96.25 ± 32.85 pg/mL) compared to the serum samples from the healthy control group (208.46 ± 58.50 pg/mL), (*p* < 0.0001), Figure 1A. Moreover, Gleason scores of 6, 7, 8, 9 and 10 regarding the prostate cancer serum samples as well as healthy control serum samples were compared (Figure 1B, Top), showing a significant decrease between the healthy control group (208.46 ± 58.50 pg/mL) and a Gleason score of 6 (14.56 ± 9.564 pg/mL; *p* = 0.0061), Figure 1B (Bottom). The total HAVcR-1 protein expression was then assessed to investigate the relationship between the total expression and serum ectodomain levels. To achieve this, as well as to investigate the localisation of HAVCR-1 in prostate tissue, total HAVcR-1 in prostate cancer (*n* = 2) and background control (*n* = 2) tissue samples were stained via IHC, Figure 1C. This revealed that the HAVcR-1 protein was expressed in prostate glandular epithelia. An analysis of staining intensity, as representative of HAVcR-1 expression, revealed a significant increase in HAVcR-1 total protein expression in malignant prostate epithelia in comparison to control prostate epithelia (*p* = 0.0006). The GSE55945 GEO dataset assessed differences in gene expression between benign prostate tissue (*n* = 8) and malignant prostate tissue (*n* = 13). When utilised for HAVcR-1 gene expression, there was a significant increase in the expression in malignant prostate tissue in comparison to benign prostate tissue (*p* = 0.047) (See Figure 1D). The GSE6919 GEO dataset was used to assess for differences in HAVcR-1 gene expression between normal prostate tissue free of any pathological alteration (*n* = 18) and primary prostate tumour samples (*n* = 65). This showed an increase in HAVcR1 gene expression in primary tumours; however, significance was not reached (*p* = 0.185), Figure 1D. The conclusion was that whilst HAVcR-1 expression was increased with prostate cancer progression, the shedding of the ectodomain was reduced, indicating that an unknown mechanism was involved in preventing shedding. 

### 3.2. Expression of HAVcR-1 in Human Prostate Cancer Cells

Levels of HAVcR-1 ectodomain released from prostate cell lines in vitro were assessed to demonstrate whether they showed a similar trend to that of serum HAVcR-1 ectodomain levels. The amount of HAVcR-1 ectodomain secreted from various cell lines within 24 h was assessed via ELISA on collected cell media, as shown in Figure 1E. There was no significant difference between HAVcR-1 ectodomain levels from the cell media of PC-3 cells (0.11 ± 0.025), Du145 cells (0.11 ± 0.032), LNCaP cells (0.08 ± 0.014), CA-HPV-7 cells (0.08 ± 0.01) or PZ-HPV-7 cells (0.07 ± 0.013). The expression of HAVcR-1 mature and immature (100 kDa and 70 kDa) cellular protein levels was assessed in various prostate cell lines, alongside the HECV cell line as a positive control, using Western Blots and IF staining. Quantification was determined using ImageJ software and analysed as the fold change relative to the HECV-positive control. A similar trend was seen in the expression of both the mature and immature protein, with a greater expression in metastatic tumour-derived cell lines PC3, Du145 and LNCaP than in the immortalised cell lines CA-HPV-10 and PZ-HPV-7. The highest expression was in LNCaP cells and the lowest in PZ-HPV-7 cells; however, significance was not reached (Figure 1F,G) with staining intensity shown in Figure 1H. The total HAVcR-1 staining also showed a greater protein expression in PC3, Du145 and LNCaP cell lines than the CA-HPV-10 cell line, and this in turn was greater than the expression in the PZ-HPV-7 cell line. The HAVcR-1 gene expression of several prostate cell lines was assessed using qPCR. qPCR data were analysed as the fold change relative to the HECV-positive control. qPCR revealed a higher HAVcR-1 gene expression in PC3 cells than in LNCaP, CA-HPV-10 and PZ-HPV-7 cell lines, with the highest HAVcR-1 gene expression seen in Du145 cells and the lowest in PZ-HPV-7 cells; however, significance was not reached (Figure 1I). These data showed that unlike the patient samples, ectodomain shedding was not associated with an increased metastatic derivation of prostate cancer cell lines, although mature/immature protein expression was indeed increased in more aggressive cancer types.

### 3.3. Modulation of HAVcR-1 Expression in PC-3 Human Prostate Cancer Cells

**Figure 2:**
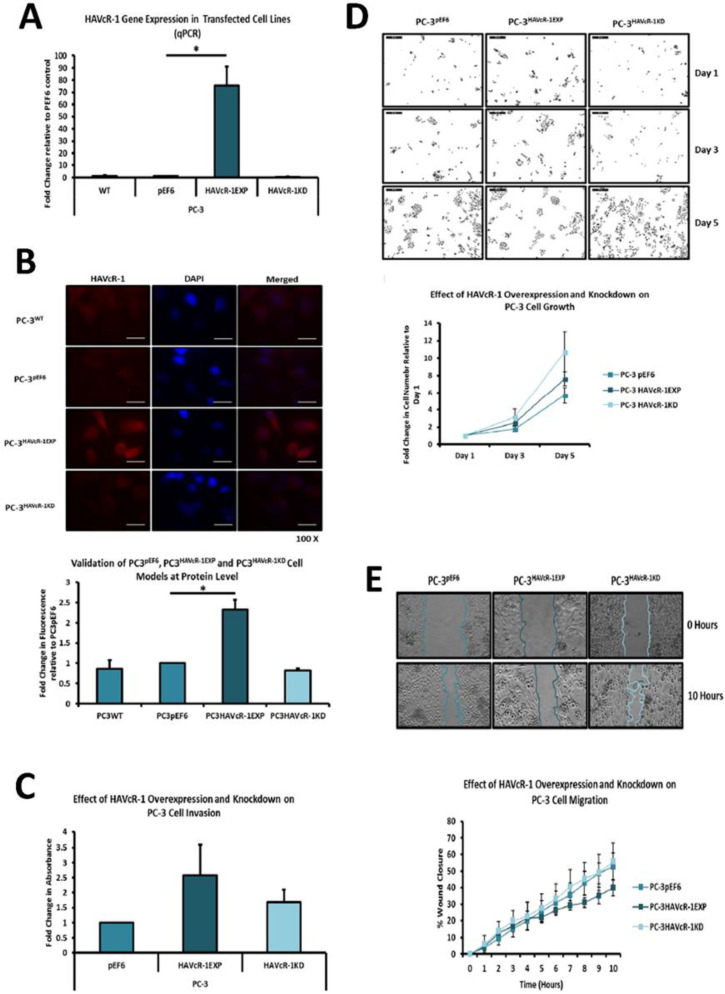
HAVcR-1 knockdown and overexpression in PC-3 prostate cancer cell line. (**A**) Confirmation of gene knockdown via qPCR. (**B**) Top shows protein validation of HAVcR-1 overexpression and knockdown in PC-3 cell lines using immunofluorescence. Images show fluorescence emission at 100× magnification correlating to HAVcR-1 expression or nuclear staining and a merged image of both. Images are representative of three independent experiments. Scale bars represent 20 µm. (**B**) Bottom graph shows quantitative analysis of immunofluorescent staining of HAVcR-1 (mean +SEM, *n* = 3, * represents *p* ≤ 0.05). Fold change was calculated using PC-3 pEF6 score of 1, as the baseline intensity. (**C**) Effect of HAVcR-1 overexpression and knockdown on PC-3 cell invasion. Graph shows the means of three independent experiments as fold change relative to the absorbance of the pEF6 control with error bars showing SEM. (**D**) Effect of HAVcR-1 overexpression and knockdown on PC-3 cell growth. Images are representative of three independent experiments. Scale bars represent 2 mm. Graph shows the means of three independent experiments as fold change relative to the cell count at day 1 with error bars showing SEM. (**E**) Effect of HAVcR-1 overexpression and knockdown on PC-3 cell migration. Images shown are representative of three independent experiments. Data shown are the means of three independent experiments and error bars represent SEM.

PC-3 cells, which were chosen for genetic modification due to the intermediate expression of HAVcR-1, were transfected via electroporation with plasmids: the pEF6 control (termed PC-3 pEF6), HAVcR-1EXP plasmid (termed PC-3 HAVcR-1EXP) or HAVcR-1KD plasmid (termed PC-3 HAVcR-1KD). The success of these transfections was assessed at the mRNA level via qPCR (Figure 2A). qPCR showed a significant increase in HAVcR-1 expression in PC-3 HAVcR-1EXP with a 75.26 ± 15.91-fold increase (*p* = 0.043). qPCR showed a 0.51 ± 0.22-fold decrease in HAVCR-1 expression in PC-3 HAVcR-1KD cells, as shown in Figure 2A. IF staining was utilised to validate successful cell transfection at the protein level (Figure 2B Top) and protein expression was quantified using ImageJ software (Figure 2B bottom). The fold change was calculated using PC-3 pEF6 as a score of one, as the baseline intensity. As expected, there was no difference in expression between PC-3 WT and PC-3 pEF6 (*p* = 0.600), but there was an increased expression of HAVcR-1 in PC-3 HAVcR-1EXP (2.32 ± 0.25 fold; *p* = 0.033). The protein expression of HAVcR-1 in PC-3 HAVcR-1KD decreased by 0.82 ± 0.05-fold from that of PC-3 pEF6; however, this did not reach significance due to the initial low levels of endogenous HAVcR-1 in PC-3 WT cells in general (*p* = 0.079). The staining of HAVcR-1 was diffuse throughout the cell. Within PC-3 HAVcR-1EXPHAVcR-1, the staining increased within the cytoplasm and nucleus, as shown in Figure 2B. The conclusion here was that we successfully overexpressed or knocked down HAVcR-1 in an aggressive prostate cancer cell line (PC-3), with an increased expression of HAVcR-1 leading to cytoplasmic and nuclear location, compared to a diffuse location in unmodified cells.

### 3.4. Effect of HAVcR-1 Modulation of Behaviour of PC-3 Human Prostate Cancer Cells

There was no significant difference in the invasive potential after the modulation of HAVcR-1, PC-3 HAVcR-1EXP versus PC-3 pEF6 (*p* = 0.26) and PC-3 HAVcR-1KD versus PC-3 pEF6 (*p* = 0.24), as shown in Figure 2C. The influence, if any, of HAVcR-1 modulation on PC-3 cell growth was assessed, with no significant change in cell growth in PC-3 HAVcR-1EXP in comparison to PC-3 pEF6 at day 3 (2.50 ± 0.66 vs. 1.52 ± 0.20: *p* = 0.40) or at day 5 (7.61 ± 0.81 vs. 5.67 ± 0.20: *p* = 0.185). It also showed no significant change in growth between HAVcR-1 PC-3 HAVcR-1KD and PC-3 pEF6 at day 3 (3.15 ± 1.00 vs. 1.52 ± 0.20: *p* = 0.29) or day 5 (10.71 ± 2.30 vs. 5.67 ± 0.20: *p* = 0.15), as shown in Figure 2D. There was also no significant change in cell adhesion in PC-3 pEF6 and PC-3 HAVcR-1EXP (*p* = 0.207) or PC-3 HAVcR-1KD (*p* = 0.250). Migration was also assessed, with no significant change in the wound healing of PC-3 HAVcR-1EXP in comparison to PC-3 pEF6 at any time point tested or difference in the healing rate with PC-3 HAVcR-1EXP closing 4.68 ± 0.57%/h and PC-3 pEF6 closing 5.01 ± 1.24%/h (*p* = 0.830). However, the linear regression revealed a significant decrease in slopes (3.68 ± 0.27 ± 5.39 ± 0.47; *p* = 0.002). There was no significant change in the wound healing of PC-3 HAVcR-1KD in comparison to PC-3 pEF6 at any time point tested and no significant difference in the healing rate with PC-3 HAVcR-1KD closing 5.88 ± 0.73%/h and PC-3 pEF6 closing 5.01 ± 1.24%/h (*p* = 0.730). The linear regression showed no significant difference between slopes (5.42 ± 0.68 ± 5.39 ± 0.47; *p* = 0.974), as shown in Figure 2E. Therefore, from these data, we found that neither the overexpression nor knockdown effected changes in cell growth, adhesion or invasiveness; however, there was a significant reduction in the speed of migration in PC-3 cells overexpressing HAVcR-1.

**Figure 3:**
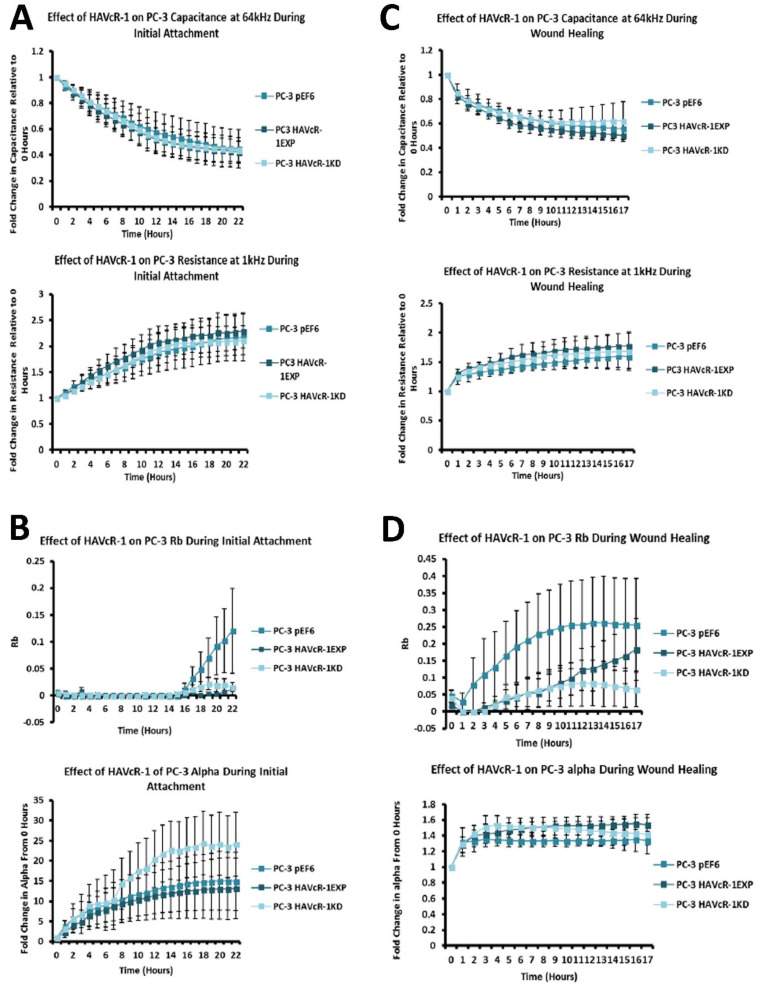
HAVcR-1 overexpression and knockdown on PC-3 initial attachment, spreading and migration. (**A**) Cells seeded in octuplicate into 96W1E+ plates at 5 × 10^4^ cells per well and resistance, capacitance and impedance were monitored for 22 h post seeding at varying frequencies ranging from 1 to 64 kHz. Graphs show the means of three independent experiments as fold change relative to 0 h with error bars showing SEM for resistance at 1 kHz and capacitance at 64 kHz. (**B**) The ECIS™ model was applied to initial attachment data to give Rb (barrier function resistance) and alpha (constraint on current flow beneath the cells) values. Graph shows the means of three independent experiments with error bars showing SEM for A Rb and B alpha shown as fold change relative to 0 h. (**C**) Post initial attachment and spreading cells were electrically wounded at 6000 Hz and 3000 μA for 30 s. Resistance, capacitance and impedance were then monitored at varying frequencies (1–64 kHz) for 17 h. Graphs show the means of three independent experiments as fold change relative to 0 h with error bars showing SEM for resistance at 1 kHz and capacitance at 64 kHz. (**D**) ECIS™ model was applied to electrical wound healing data to give Rb (barrier function resistance) and alpha (constraint on current flow beneath the cells) values. Graph shows the means of three independent experiments with error bars showing SEM for Rb and alpha shown as fold change relative to 0 h.

The cell attachment and spreading of PC-3 cells was assessed using ECIS at 64 kHz (the flow of current mainly flowed through the cells at this frequency; thus, it was representative of the amount of cell coverage on the electrode); no change in capacitance was observed in PC-3 HAVcR-1EXP and PC-3 HAVcR-1KD versus PC-3 pEF6 during PC-3 initial attachment and spreading, as shown in Figure 3A (top). The linear regression analysis showed no significance. Resistance at 1 kHz was also assessed (at this frequency, the current mainly flowed outside the cell and, therefore, was representative of cellular interactions with both the electrode as well as with adjacent cells). Again, no change was observed, as shown in Figure 3A (bottom). When assessing the barrier function using Rb modelling, no significant change in Rb was observed at any time point, as shown in Figure 3B (top). However, the linear regression analysis showed significant decreases between the slopes of PC-3 HAVcR-1EXP (0.0001 ± 0.0001) and PC-3 HAVcR-1KD (0.0008 ± 0.0002) in comparison to PC-3 pEF6 (0.0042 ± 0.0001) (*p* ˂ 0.0001 and *p* ˂ 0.0001, respectively). The adhesion to the electrode was also assessed via alpha (constraint on current flow beneath the cells), which also showed no significant change any time point, as shown in Figure 3B (bottom). There was a significant difference between the slopes of PC-3 HAVcR-1KD (1.094 ± 0.177) and PC-3 pEF6 (0.552 ± 0.087) (*p* = 0.007, but not between PC-3 HAVcR-1EXP and PC-3 pEF6 (0.552 ± 0.087) (*p* = 0.225). These data showed that there was a reduction in the cell movement of the cells overexpressing HAVcR-1, which concurred with more traditional migration assays. We also noted a significant difference in the alpha measurements in knockdown cells, indicating that a reduction in HAVcR-1 expression led to the prevention of current movement beneath the cells. 

ECIS-based cell migration was utilised, with capacitance at 64 kHz measured for 17 h post wounding; there was little change with PC-3 HAVcR-1EXP or PC-3 HAVcR-1KD versus PC-3 pEF6 during wound healing, as shown in Figure 3C (top), even after the linear regression. Resistance at 1 kHz was also measured to assess the cell–cell and cell–plate interactions; there was no change in resistance with PC-3 HAVcR-1EXP and PC-3 HAVcR-1KD versus PC-3 pEF6 during wound healing, as shown in Figure 3C (bottom); again, even when using linear regression. There was also no significance in the barrier function (Rb and Alpha). Using linear regression, there was, however, a significant increase in the slope of PC-3 HAVcR-1EXP (0.018 ± 0.003) versus PC-3 pEF6 (0.006 ± 0.003) (*p* = 0.003) when assessing the Rb barrier function, as shown in Figure 3D (top), but not alpha, as shown in Figure 3D (bottom). From these data, we concluded that HAVcR-1 knockdown did not affect changes in the barrier function whereas there was an increase in the Rb barrier function in PC-3 cells overexpressing HAVcR-1, indicating an increased cell–cell adhesion.

### 3.5. HAVcR-1 Expression and Tight Junction Protein Expression and Function in PC-3 Human Prostate Cancer Cells

**Figure 4:**
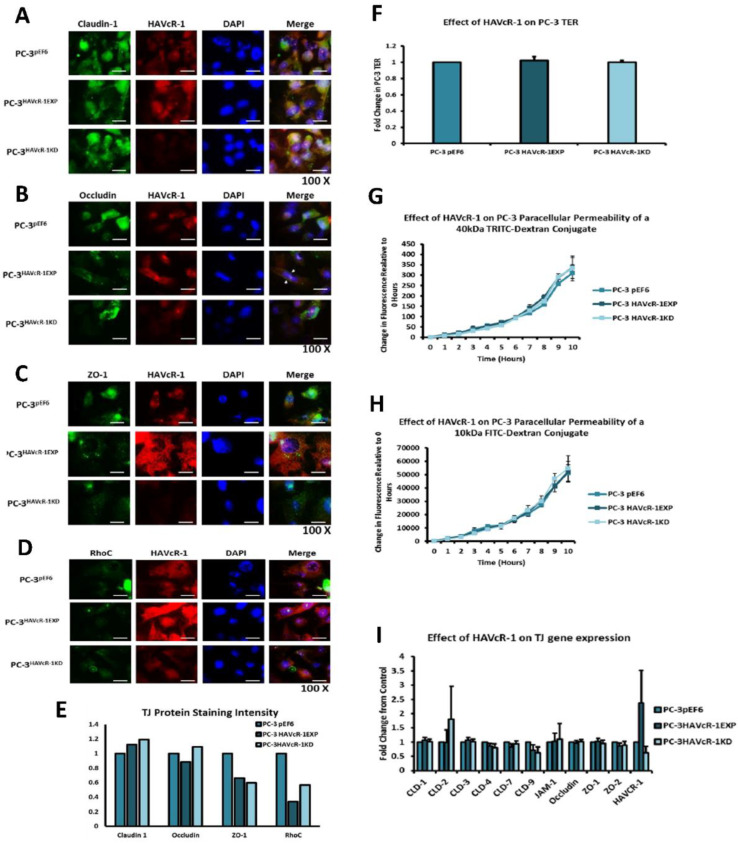
Analysis of TJ molecule expression after knockdown of HAVcR-1 in PC-3 prostate cancer cells. (**A**–**D**) Effect of HAVcR-1 on TJ protein expression and localisation at 100% confluence. Data shown are of *n* = 1. Images show fluorescence emission correlating to protein expression ((**A**) Claudin 1, (**B**) occludin, (**C**) ZO-1 and (**D**) RhoC), HAVcR-1 expression, DAPI nuclear staining and a merged image of both. Images were taken at 100× magnification and scale bars represent 20 µm. (**E**) Graph shows quantitative analysis of immunofluorescent staining of proteins. White arrows highlight membranous staining. (**F**) Transepithelial resistance. Post incubation resistance across the membrane was measured. Graph shows the means of three independent experiments as fold change relative to the resistance of PC-3 pEF6. Error bars show SEM. (**G**) Paracellular permeability. Post incubation cell media inside of the inserts were changed to media containing 0.2 mg/mL of 40 kDa TRITC–dextran conjugate and 0.2 mg/mL of 10 kDa FITC–dextran conjugate. Graph shows the means of three independent experiments as change to fluorescence from 0 h for 40 kDa TRITC–dextran conjugate to 10 kDa FITC–dextran conjugate (**H**). Error bars show SEM. (**I**) Gene expression of TJ proteins. Data shown are the means of three independent experiments and error bars show SEM. Graph shows band intensity as quantified by ImageJ software. Expression was normalised to GAPDH and is shown as fold change relative to pEF6 control.

As RB (cell–cell adhesion contacts) increased with HAVcR-1 overexpression, we decided to explore the potential relationship between HAVcR-1 and TJs in PC-3 cells as they have been well characterised and we had already genetically modified the cells. Preliminary investigations into Claudin1, Occludin, ZO-1 and RhoC protein expression and localisation were assessed using immunofluorescence. PC-3 HAVcR-1EXP and PC-3 HAVcR-1KD showed increased Claudin1 versus PC-3 pEF6 cells, as shown in Figure 4A. The staining of Claudin 1 was highly localised within the cytoplasm with a minority of staining at the cell membrane, whilst it was diffuse throughout the cell and showed no change with regards to the manipulation of HAVcR-1. The occludin staining intensity decreased in PC-3 HAVcR-1EXP cells and increased in PC-3 HAVcR-1KD cells versus PC-3 pEF6 cells, as shown in Figure 4B. PC-3 pEF6 and PC-3 HAVcR-1KD cells had diffuse staining throughout the cell, although with PC-3 HAVcR-1EXP cells, the staining intensity decreased, and there was clear staining at the cell membrane. The ZO-1 staining intensity decreased in both PC-3 HAVcR-1EXP and PC-3 HAVcR-1KD cells versus PC-3 pEF6 cells, with diffuse staining throughout the cell in all cases with some decreased nuclear staining within PC-3 HAVcR-1EXP cells, as shown in Figure 4C. The RhoC staining intensity also decreased in both PC-3 HAVcR-1EXP and PC-3 HAVcR-1KD cells versus PC-3 pEF6 cells. The expression of HAVcR-1 had no effect on the localisation of RhoC, as shown in Figure 4D. This was summarised using fold change in Figure 4E. We concluded from these data that the modulation of HAVcR-1 caused changes in three TJ proteins. Claudin1 and occludin, two transmembrane proteins, showed that expression increased with a reduced HAVcR-1. Occludin reduced with the overexpression of HAVcR-1. ZO-1, a TJ anchoring protein, reduced in both cases, as was the signalling protein RhoC. TJ has an enormous complexity, but these data indicated that the knockdown of HAVcR-1 caused an increased transmembrane protein expression, agreeing with the increased Rb function observed in Figure 3C,D.

We assessed any changes in TJ function using TER and PCP. Using an in vitro TER assay, we found no significant change in PC-3 HAVcR-1EXP, with a 1.02 ± 0.05-fold change versus the PC-3 pEF6 control (*p* = 0.706), as shown in Figure 4F. There was also no significant change in PC-3 HAVcR-1KD, with a 1.00 ± 0.02-fold change versus PC-3 pEF6 cells (*p* = 0.999). The in vitro PCP assay revealed no change in the amount of 40 kDa TRITC–dextran conjugate able to pass through PC-3 HAVcR-1EXP monolayers versus PC-3 pEF6 monolayers with no significant difference at any time point, and the linear regression analysis showed no significant difference between slopes (32.25 ± 2.63 vs. 28.66 ± 2.27; *p* = 0.304), as shown in Figure 4G, nor in PC-3 HAVcR-1KD versus PC-3 pEF6 monolayers (32.09 ± 2.77 vs. 28.66 ± 2.27; *p* = 0.341). There was also no change in the passage of a smaller 10 kDa FITC–dextran conjugate in PC-3 HAVcR-1EXP versus PC-3 pEF6 monolayers (4871 ± 374.7 vs. 4717 ± 343.8; *p* = 0.762), as shown in Figure 4H, or PC-3 HAVcR-1KD versus PC-3 pEF6 monolayers (5257 ± 453.7 vs. 4717 ± 343.8; *p* = 0.346. The barrier function is a composite of a number of adhesion factors, beneath cells, through cells and between cells. These data reinforced our findings that HAVcR-1 is associated with changed in the Rb barrier function, i.e., between cells only.

We also investigated changes in the gene expression of ten TJ proteins in PC-3 HAVcR-1EXPand PC-3 HAVcR-1KD cells versus expression in PC-3 pEF6. From these ten genes, eight encoded integral membrane proteins (Claudin-1, -2, -3, -4, -7 and -9, occludin and JAM-1) and two encoded plaque anchoring proteins (ZO -1 and ZO-2) were determined. There was no significant change in the expression in PC-3 HAVcR-1EXP versus PC-3 pEF6 for Claudin-1, Claudin-2, Claudin-3, Claudin-4, Claudin-7, Claudin-9, JAM-1, occludin, ZO-1 or ZO-2 (*p* > 0.05). There was also no significant change in the gene expression in PC-3 HAVcR-1KD in comparison to PC-3 pEF6, as shown in Figure 4I. In comparison to the protein staining, there was no effect on the gene expression of TJ molecules after HAVcR-1 modulation, indicating that any effects were post-translational and more likely to be due to regulatory pathways.

### 3.6. Modulation of HAVcR-1 Expression in PZ-HPV-7 Human Prostate Cancer Cells

**Figure 5:**
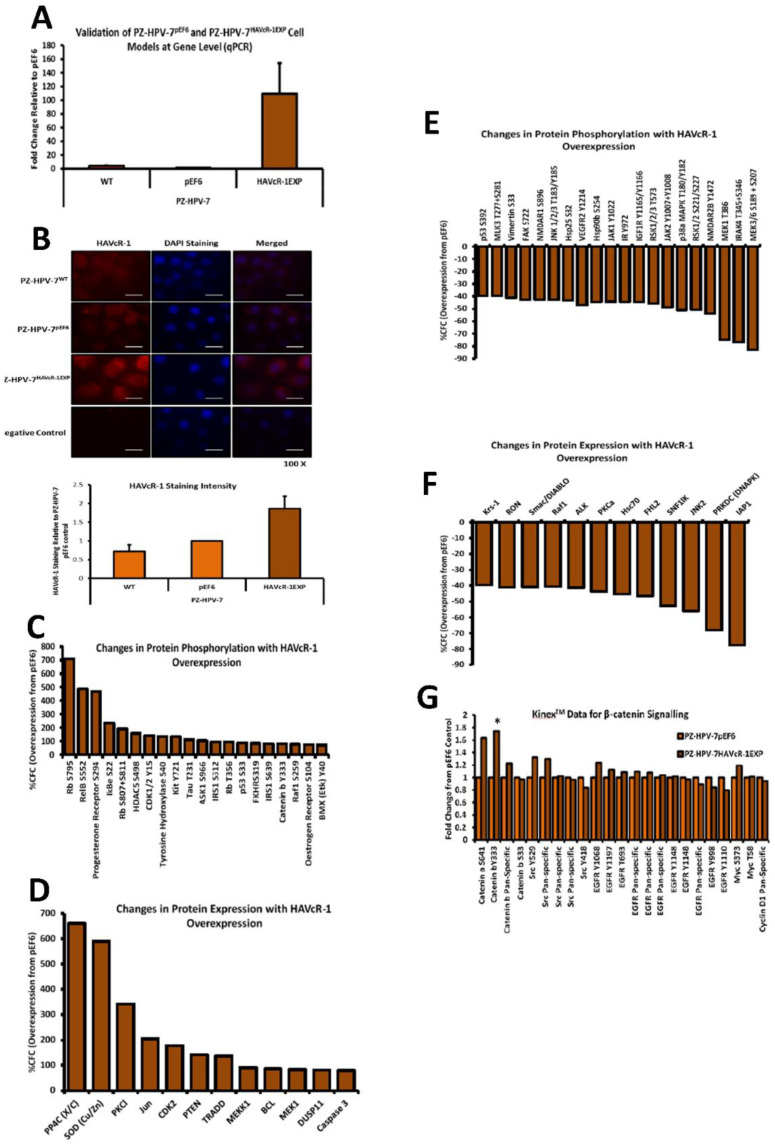
HAVcR-1 overexpression in PZ-HPV-7 prostate cancer cell line. (**A**) Confirmation of gene knockdown via qPCR. (**B**) Protein expression validation of PZ-HPV-7 pEF6 and PZ-HPV-7 HAVCR-1EXP. Images show fluorescence emission at 100× magnification correlating to HAVcR-1 expression or nuclear staining and a merged image of both. Images are representative of three independent experiments. Scale bars represent 20 µm. Graph shows quantitative analysis of immunofluorescent staining of HAVcR-1 (mean + SEM, *n* = 3). (**C**,**D**) Protein expression and phosphorylation significantly increased with HAVcR-1 overexpression. Protein was extracted from PZ-HPV-7 pEF6 and PZ-HPV-7HAVcR-1 EXP and sent to Kinex Bioinformatics for a Kinex ™ antibody microarray. Graphs show the percentage change from control of protein phosphorylation or total protein expression for all significantly increased results (z value ≥ 1.65). (**E**,**F**) Protein expression and phosphorylation significantly decreased with HAVcR-1 overexpression, (z value ≤ −1.65). (**G**) β-Catenin Y333 signalling changes with Kinex™ Antibody Microarray, showing fold change from PZ-HPV-7 pEF6 control of all proteins and phosphosites involved with β-catenin signalling included within the Kinex™ antibody microarray. * represents *p* ≤ 0.05.

Similar experiments were than carried out in non-aggressive prostate cancer cells to observe any differences to the more aggressive PC-3 cells. We hoped to discover any changes in phospho proteins after HAVcR-1 overexpression. PZ-HPV-7 cells, which had little HAVcR-1 endogenous expression, were successfully transfected with the pEF6 control plasmid to form PZ-HPV-7 pEF6 or the HAVcR-1EXP plasmid to form PZ-HPV-7 HAVcR-1EXP, as shown in Figure 5A. Immunofluorescence assessed the HAVcR-1 protein expression and further validated the PZ-HPV-7 pEF6 and PZ-HPV-7 HAVcR-1EXP cell models, Figure 5B (Top). Cells were stained for total HAVcR-1 as well as for the nucleus using DAPI staining and quantified using ImageJ software and analysed as the fold change relative to PZ-HPV-7 pEF6 HAVcR-1 expression. There was an increase in the HAVcR-1 protein expression in PZ-HPV-7 HAVcR-1EXP, with a 1.86 ± 0.58-fold increase from the PZ-HPV-7 pEF6 HAVcR-1 protein expression, although significance was not reached (*p* = 0.375), as shown in Figure 5B (bottom).

### 3.7. Results from Kinexus Analysis of Phosphorylated Proteins after Induced Expression of HAVcR-1 in PZ-HPV-7 Human Prostate Cancer Cells

Kinex™ KAM-880 antibody microarrays were used on protein lysates extracted from PZ-HPV-7 pEF6 and PZ-HPV-7 HAVcR-1EXP cells. This showed 20 significantly increased phosphorylations at specific phosphosites in PZ-HPV-7 HAVcR-1EXP versus PZ-HPV-7 pEF6, as shown in Figure 5C. It also showed the total expression of 12 proteins, which were significantly increased, as shown in Figure 5D. There were also 20 cases of a decreased phosphorylation at specific phosphosites in PZ-HPV-7 HAVcR-1EXP versus PZ-HPV-7 pEF6, as shown in Figure 5E, with the total protein expression having decreased in 12 cases, as shown in Figure 5F. 

**Figure 6:**
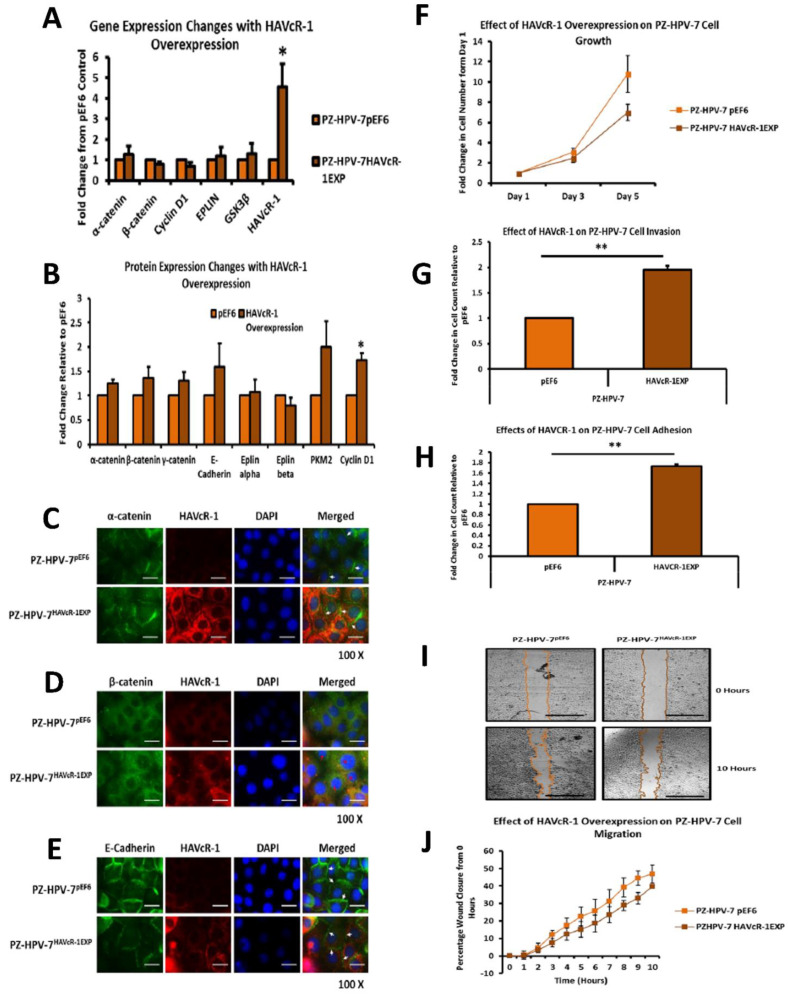
HAVcR-1 overexpression in PZ-HPV-7 prostate cancer cell line and changes in β-catenin complex protein expression and cell behaviour. (**A**) Changes to α- and β-catenin signalling gene expression. HAVcR-1 mRNA expression was assessed using PCR; figure is representative of three independent experiments showing band intensity as quantified by ImageJ software. Data shown are the means of three independent experiments with gene expression shown as normalised to GAPDH and relative to PZ-HPV-7 pEF6 and error bars show SEM. (**B**) HAVcR-1 induced changes to β-catenin signalling protein expression; means of three independent experiments and error bars show SEM. Protein expression was assessed using SDS PAGE and Western blot analysis, where the graph shows band intensity as quantified by ImageJ software and normalised to GAPDH and is shown as fold change relative to PZ-HPV7 pEF6. (**C**–**E**) HAVcR-1 induced changes to α-catenin, β-catenin and E-Cadherin protein localisation; 100× magnification with (**C**) α-catenin, (**D**) β-catenin or (**E**) E-cadherin alongside HAVcR-1 expression, nuclear staining and a merged image of both. Scale bars represent 20 µm and membranous staining and nuclear staining are highlighted by white and red arrows, respectively. (**F**) HAVcR-1 overexpression on PZ-HPV-7 cell growth. Graph shows the means of three independent experiments as fold change relative to the cell count at day 1 with error bars showing SEM. (**G**) PZ-HPV-7 cell invasion, graph shows the means of three independent experiments as fold change relative to the cell count of PZ-HPV-7 pEF6 with error bars showing SEM. (**H**) PZ-HPV-7 cell adhesion, graph shows the means of three independent experiments as fold change relative to the cell count of the PZ-HPV-7 pEF6 control with error bars showing SEM. (**I**,**J**) HAVcR-1 overexpression on PZ-HPV-7 cell migration. Images taken at 5× magnification immediately afterward and every hour thereafter. Images shown are representative of three independent experiments. Scale bars represent 2 mm. Wound area was measured using ImageJ software and percentage wound closure was calculated as relative to 0 h time point. Data shown are the means of three independent experiments and error bars represent SEM. Significance is indicated by * and **, which signify *p* ≤ 0.05 and *p* ≤ 0.01, respectively.

Drilling down into the data, β-catenin showed a 1.74-fold increase at the Y333 phosphorylation site in PZ-HPV-7 HAVcR-1EXP when compared to levels in PZ-HPV-7 pEF6 (z value = 1.77) and was chosen for further study, as shown in Figure 5G. There was also a 1.63-fold increase in α-catenin S641 phosphorylation; however, this was not significant. Subsequently, we assessed array data for proteins involved in the β-catenin Y333 signalling pathway, including Src, EGFR, c-Myc and cyclin D1, as shown in Figure 5G; the only significant change was that of β-catenin Y333 phosphorylation. There was also no significant change in the expression of α-catenin (1.28 ± 0.40; *p* = 0.470), β-catenin (0.78 ± 0.15; *p* = 0.177), cyclin D1 (0.70 ± 0.18; *p* = 0.161), EPLIN (1.21 ± 0.70; *p* = 0.591) or GSKβ (1.30 ± 0.51; *p* = 0.541) in PZ-HPV-7 HAVcR-1EXP versus PZ-HPV-7 pEF6. This was outlined in the schematic, as shown in Figure 5I. There was no significant change in the protein expression of α-catenin (1.26 ± 0.07; *p* = 0.073), β-catenin (1.37 ± 0.22; *p* = 0.243), E-Cadherin (1.59 ± 0.487; *p* = 0.352), EPLIN-β (1.07 ± 0.27; *p* = 0.82), EPLIN-α (0.79 ± 0.16; *p* = 0.339) or PKM2 (2.00 ± 0.53; *p* = 0.199) in PZ-HPV-7 HAVcR-1EXP versus PZ-HPV-7 pEF6. There was a significant increase in cyclin D1 protein expression by 1.74 ± 0.13; *p* = 0.030 in PZ-HPV-7 HAVcR-1EXP versus PZ-HPV-7 pEF6, as shown in Figure 6A,B. The localisation of α-catenin, β-catenin and E-cadherin was also assessed via immunofluorescence. This showed a potential increased membrane localisation of α-catenin, although staining was discontinuous (see Figure 6C), an increased nuclear localisation of β-catenin (see Figure 6D) and a decreased membrane localisation of E-cadherin, as shown in Figure 6C–E. The overexpression of HAVcR-1 appeared to affect changes in the phosphorylation status of α and β-catenins, both of which are involved in cell motility and are part of the complex involved in HGF signalling, a key factor in the metastatic behaviour of cancer cells.

### 3.8. Effect of HAVcR-1 Modulation of Behaviour of PZ-HPV-7 Human Prostate Cancer Cells

PZ-HPV-7 HAVcR-1EXP and PZ-HPV-7 pEF6 were used to assess the effect that HAVcR-1 expression has on cell proliferation. There was no significant difference in cell growth with PZ-HPV-7 HAVcR-1EXP versus PZ-HPV-7 pEF6 at day 3 (2.47 ± 0.45 vs. 3.10 ± 0.36; *p* = 0.34) or at day 5 (7.00 ± 0.8 vs. 10.8 ± 1.82; *p* = 0.16), as shown in Figure 6F. When assessing the invasive potential, we saw an increase in the cell invasion with PZ-HPV-7 HAVcR-1EXP versus PZ-HPV-7 pEF6, with a 1.95 ± 0.07-fold increase (*p* = 0.006), as shown in Figure 6G. An adhesion assay revealed a significant 1.73 ± 0.04-fold increase in PZ-HPV-7 HAVcR-1EXP versus PZ-HPV-7 pEF6 (*p* = 0.002), as shown in Figure 6H. Migration assays showed no significant difference in wound healing at any time point within 10 h between the two cell lines, as shown in Figure 6I,J. There was also no significant difference in the healing rate with PZ-HPV-7 HAVcR-1EXP closing 3.53 ± 0.42%/h in comparison to PZ-HPV-7 pEF6 closing 4.64 ± 0.70%/h (*p* = 0.262), and the linear regression analysis showed a significant decrease between the slope of PZ-HPV-7 HAVcR-1EXP (4.04 ± 0.27) and PZ-HPV-7 pEF6 (5.10 ± 0.37) (*p* = 0.025). From these data, we concluded that the overexpression of HAVcR-1 in non-aggressive prostate cancer cells led to the acquirement of an invasive phenotype.

### 3.9. HAVcR-1 Expression, Barrier Function and Tight Junction Protein Expression and Function in PZ-HPV-7 Human Prostate Cancer Cells

**Figure 7:**
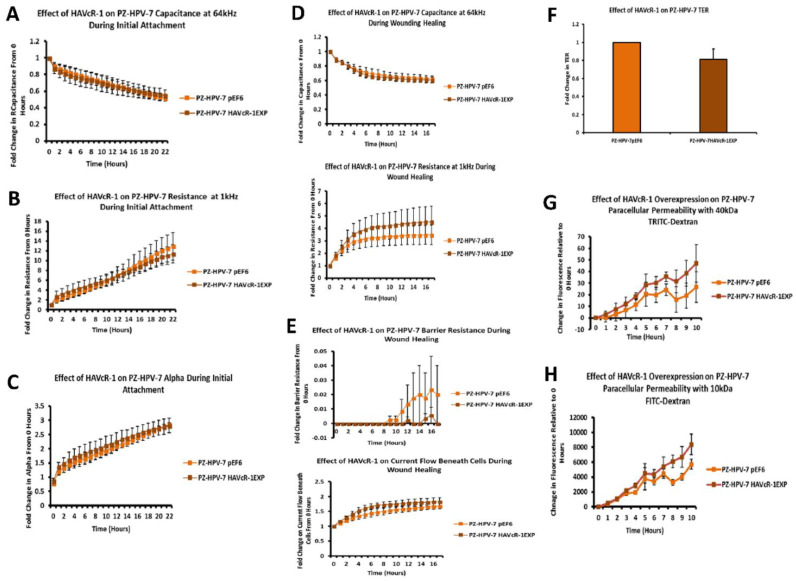
HAVcR-1 overexpression in PZ-HPV-7 prostate cancer cell line and initial attachment, spreading, migration and barrier function. (**A**,**B**) Effects of HAVcR-1 overexpression on PZ-HPV-7 initial attachment and spreading, resistance, capacitance and impedance were monitored for 22 h post seeding at varying frequencies ranging from 1 to 64 kHz. Graphs show the means of three independent experiments as fold change relative to 0 h with error bars showing SEM for A capacitance at 64 kHz and B resistance at 1 kHz. (**C**) Constraint on current flow beneath PZ-HPV-7 cells during initial attachment and spreading. The ECIS™ model was applied to initial attachment data using the ECIS software to give alpha values. Graph shows the means of three independent experiments with error bars showing SEM for alpha shown as fold change relative to 0 h. (**D**) Electrical wound healing post initial attachment and spreading cells were electrically wounded at 6000 Hz and 3000 μA for 30 s. Resistance, capacitance and impedance were then monitored at varying frequencies (1–64 kHz) for 17 h. Graphs show the means of three independent experiments as fold change relative to 0 h with error bars showing SEM for A resistance at 1 kHz and B capacitance at 64 kHz. (**E**) Current flow beneath PZ-HPV-7 cells and barrier resistance during electrical wound healing. The ECIS™ model was applied to wound healing data using the ECIS software to give Rb (barrier resistance) and alpha values. Graphs show the means of three independent experiments with error bars showing SEM shown as fold change relative to 0 h. (**F**) Transepithelial resistance (TER) graph shows the means of three independent experiments as fold change relative to PZ-HPV-7 pEF6. Error bars show SEM. (**G**,**H**) Paracellular permeability (PCP) graphs showing the means of three independent experiments as change in fluorescence from 0 h of A the 40 kDa TRITC–dextran conjugate to B the 10 kDa FITC–dextran conjugate. Error bars show SEM.

ECIS was utilised to investigate the effect of HAVcR-1 on PZ-HPV-7 initial attachment and spreading at a capacitance of 64 kHz. There was no change in PZ-HPV-7 HAVcR-1EXP versus PZ-HPV-7 pEF6, as shown in Figure 7A. The linear regression analysis also showed no significant difference for PZ-HPV-7 HAVcR-1EXP (−0.016 ± 0.002) and PZ-HPV-7 pEF6 (−0.019 ± 0.001) (*p* = 0.336). Resistance at 1 kHz also provided no change in resistance between PZ-HPV-7 HAVcR-1EXP and PZ-HPV-7 pEF6, as shown in Figure 7B, as did the linear regression analysis, PZ-HPV-7 HAVcR-1EXP (0.43 ± 0.03) and PZ-HPV-7 pEF6 (0.54 ± 0.06) (*p* = 0.112). Alpha (constraint on current flow beneath cells) modelling showed no difference between PZ-HPV-7 HAVcR-1EXP and PZ-HPV-7 pEF6, as shown in Figure 7C, with no significant difference using linear regression, PZ-HPV-7 HAVcR-1EXP (0.09 ± 0.01) and PZ-HPV-7 pEF6 (0.10 ± 0.01) (*p* = 0.334). 

Electrical wounding was assessed using capacitance at 64 kHz and demonstrated no difference in capacitance between PZ-HPV-7 HAVcR-1EXP and PZ-HPV-7 pEF6, as shown in Figure 7D (top), nor using the linear regression analysis, PZ-HPV-7 HAVcR-1EXP (−0.019 ± 0.002) and PZ-HPV-7 pEF6 (−0.018 ± 0.002) (*p* = 0.778). For changes in junctional complexes, resistance at 1 kHz was assessed and, again, there was no difference between PZ-HPV-7 HAVcR-1EXP and PZ-HPV-7 pEF6, as shown in Figure 7D (bottom), linear regression PZ-HPV-7 HAVcR-1EXP (0.16 ± 0.04) and PZ-HPV-7 pEF6 (0.11 ± 0.03) (*p* = 0.264). There was also no significant change in either alpha or Rb with PZ-HPV-7 HAVcR-1EXP versus PZ-HPV-7 pEF6, as shown in Figure 7E.

TER data were analysed as the fold change from the PZ-HPV-7 pEF6, with no significant change in the TER of PZ-HPV-7 HAVcR-1EXP, with a 0.81 ± 0.12-fold change in comparison to the PZ-HPV-7 pEF6 control (*p* = 0.248), as shown in Figure 7F. Moreover, there was no significant difference between the PCP of PZ-HPV-7 HAVcR-1EXP and PZ-HPV-7 pEF6, as shown in Figure 7G,H. The linear regression analysis of 40 kDa dextran PCP revealed a significant increase in the slopes of PZ-HPV-7 HAVcR-1EXP (4.62 ± 0.66) versus PZ-HPV-7 pEF6 (2.64 ± 0.68) (*p* = 0.042), and when analysing the 10 kDa dextran PCP, it also showed a significant increase in the slopes of PZ-HPV-7 HAVcR-1EXP (816.50 ± 72.85) versus PZ-HPV-7 pEF6 (516.30 ± 57.31). From the barrier function assessment, the overexpression of HAVcR-1 in non-aggressive prostate cancer cells led to little difference in the physical barrier function (TER/Rb/alpha), but there was a significant increase in the paracellular permeability, indicating a reduction in a robust extracellular route, allowing the passage of substances of all sizes.

### 3.10. Assessment of the Action of HGF on Human Prostate Cell Behaviour with Modulated HAVcR-1 Expression

**Figure 8:**
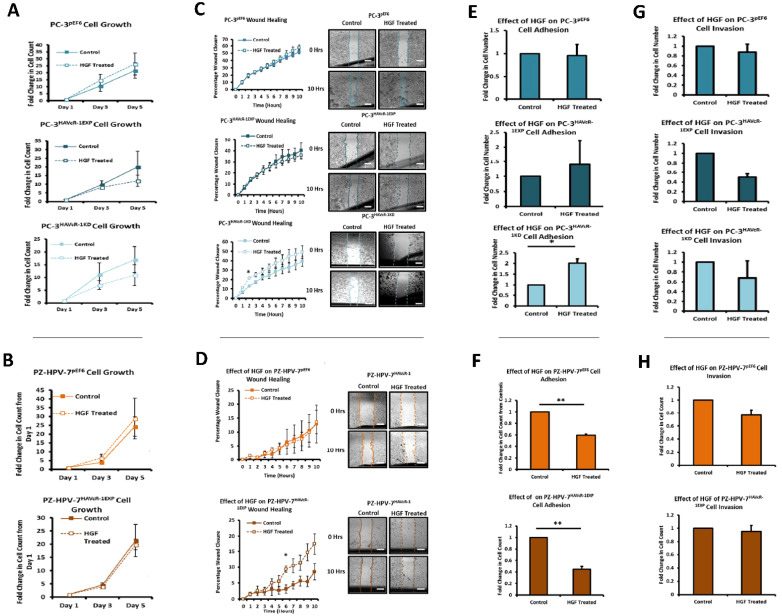
Comparison of HAVcR-1 expression modulation and treatment with HGF (40 ng/mL) on prostate cancer cell behaviour. (**A**) HAVcR-1 in combination with HGF on PC-3 cell growth. Scale bars are representative of 2 mm and graphs show the means of three independent experiments as fold change relative to the cell count at day 1 with error bars showing SEM. (**B**) HAVcR-1 in combination with HGF on PZ-HPV-7 cell growth. (**C**) HAVcR-1 in combination with HGF on PC-3 cell migration; images were taken immediately afterward and every hour thereafter at 5 X magnification. Wound area was measured using ImageJ software and percentage wound closures were calculated as relative to 0 h time point. Data shown are the means of three independent experiments and error bars represent SEM. Images shown are representative of three independent experiments and scale bars are representative for 2 mm. (**D**) HAVcR-1 in combination with HGF on PZ-HPV-7 cell migration, images were taken immediately afterward and every hour thereafter at 5 X magnification. (**E**) HAVcR-1 in combination with HGF on PC-3 cell adhesion; graphs show the means of three independent experiments as fold change relative to the cell count of the control with error bars showing SEM. (**F**) HAVcR-1 in combination with HGF on PZ-HPV-7 cell adhesion. (**G**) HAVcR-1 in combination with HGF on PC-3 cell invasion, graphs show the means of three independent experiments as fold change relative to controls with error bars showing SEM. (**H**) HAVcR-1 in combination with HGF on PZ-HPV-7 cell invasion, graphs show the means of three independent experiments as fold change relative to controls with error bars showing SEM. Significance is indicated by * and **, which signify *p* ≤ 0.05 and *p* ≤ 0.01, respectively.

Given the data indicating the involvement of the catenin complex in HAVcR-1 expression, PC-3 pEF6 and PZ-HPV-7 pEF6 cell models were utilised to assess the effect of HGF on cell growth, migration and adhesion. We also used PC-3 HAVcR-1EXP, PC-3 HAVcR-1KD and PZ-HPV-7 HAVcR-1EXP cells to assess whether HAVcR-1 influenced these HGF-induced changes to cell growth. The cell growth exhibited no significant difference with the HGF treatment of PC-3 pEF6 cells versus control at day 3 (14.70 ± 4.13 vs. 10.56 ± 3.93; *p* = 0.508) or at day 5 (26.36 ± 7.86 vs. 21.65 ± 5.55; *p* = 0.652), as shown in Figure 8A. HAVcR-1 overexpression had no effect on the HGF treatment of PC-3 HAVcR-1EXP cells versus control cells at day 3 (8.27 ± 1.18 vs. 9.85 ± 2.16; *p* = 0.567) or at day 5 (11.99 ± 3.21 vs. 20.07 ± 9.11; *p* = 0.476). HAVcR-1 knockdown also effected no change in cell growth with the HGF treatment of PC-3 HAVcR-1EXP versus control cells at day 3 (7.03 ± 1.76 vs. 11.23 ± 4.50; *p* = 0.457) or at day 5 (10.92 ± 4.13 vs. 17.00 ± 5.10; *p* = 0.409), Figure 8A. The HGF treatment also had no effect on PZ-HPV-7 pEF6 cell growth versus control cells at day 3 (3.21 ± 0.02 vs. 3.54 ± 0.01; *p* = 0.214) or at day 5 (7.42 ± 0.73 vs. 8.88 ± 2.32; *p* = 0.274), as shown in Figure 8B (top) nor did HAVcR-1 overexpression in PZ-HPV-7 cells with HGF treatment at day 3 (3.04 ± 0.09 vs. 3.10 ± 0.18; *p* = 0.789) or at day 5 (6.76 ± 1.13 vs. 6.55 ± 1.16; *p* = 0.905), as shown in Figure 8B (bottom). Therefore, we concluded that HGF/HAVcR-1 had no effect on prostate cancer cell growth.

The effect of HAVcR-1 on HGF-induced changes on cell migration was also assessed via the use of PC-3 HAVcR-1EXP, PC-3 HAVcR-1KD and PZ-HPV-7 HAVcR-1EXP cells; there were no significant differences in percentage wound closure at any time point or in the healing rate of PC-3 pEF6 with the HGF treatment (7.19 ± 0.78%/h) versus control (6.92 ± 0.63%/h) (*p* = 0.79), and no significant difference between slopes was detected (5.41 ± 0.30 vs. 4.76 ± 0.26; *p* = 0.103), as shown in Figure 8C. There were no significant differences with the HGF treatment of PC-3 HAVcR-1EXP with the HGF treatment (5.24 ± 0.25%/h) of PC-3 HAVcR-1EXP cells versus control cells (5.19 ± 0.63%/h) (*p* = 0.943), and no significant difference between slopes (3.33 ± 0.27 vs. 3.95 ± 0.39; *p* = 0.191). There was, however, an initial effect with a significant increase in the percentage wound closure with the HGF treatment in PC-3 HAVcR-1KD cells versus control cells at 2 h (21.59 ± 0.66 vs. 13.00 ± 0.75; *p* = 0.001). The HGF treatment also had little effect on PZ-HPV-7 pEF6 cells versus control cells at any time point or in the healing rate with the HGF-treated closing 0.95 ± 0.23%/h and control cells closing 0.76 ± 0.33%/h (*p* = 0.667), and no significant difference between slopes (1.21 ± 0.18 vs. 1.34 ± 0.32; *p* = 0.728), as shown in Figure 8D (top). However, the HGF treatment in PZ-HPV-7 HAVcR-1EXP cells increased the percentage wound closure versus control PZ-HPV-7 HAVcR-1EXP at 6 h (9.37 ± 0.98 vs. 3.12 ± 1.28; *p* = 0.020), and a significant difference between slopes (1.69 ± 0.16 vs. 0.66 ± 0.12, *p* ˂ 0.0001) was observed, as shown in Figure 8D (bottom). It appeared, therefore, that HGF induced faster migration in PC-3 cells with HAVcR-1 knockdown, whereas HGF caused an increase in the migration of PZ-HPV-7 cells overexpressing HAVcR-1. Cell adhesion assays showed no significant difference in cell adhesion with the HGF treatment of PC-3 pEF6 versus control (1.73 ± 0.04-fold increase; *p* = 0.875), as shown in Figure 8E, and HAVcR-1 overexpression in PC-3 cells had no effect with the HGF treatment of PC-3 HAVcR-1EXP, resulting in a 1.28 ± 0.68-fold increase versus control with *p* = 0.724. The HAVcR-1 knockdown in PC-3 cells did show a significantly increased adhesion of 2.05 ± 0.21-fold with the HGF treatment versus control with *p* = 0.039, as shown in Figure 8E. In PZ-HPV-7 cells, a significant decrease in cell adhesion with HGF was seen in PZ-HPV-7 pEF6 versus control (0.60 ± 0.02-fold change; *p* = 0.002), as shown in Figure 8F. When assessing cell invasion, HAVcR-1 overexpression had no significant effect with the HGF treatment, resulting in a 0.83 ± 0.15-fold change from control with *p* = 0.268 in PC-3 HAVcR-1EXP cells, as shown in Figure 8G. The havcr-1 knockdown also had no significant effect with the HGF treatment, resulting in a 1.04 ± 0.28-fold change (*p* = 0.905) from control in PC-3 HAVcR-1KD cells, as shown in Figure 8G. HAVcR-1 overexpression in PZ-HPV-7 cells had no effect on invasion after the HGF treatment of PZ-HPV-7 HAVcR-1EXP, resulting in a 0.95 ± 0.09-fold change in invasion from the control with *p* = 0.645, as shown in Figure 8H.

### 3.11. Assessment of the Action of HGF on the Modulation of HAVcR-1 Expression, Barrier Function and Tight Junction in Human Prostate Cancer Cells

**Figure 9:**
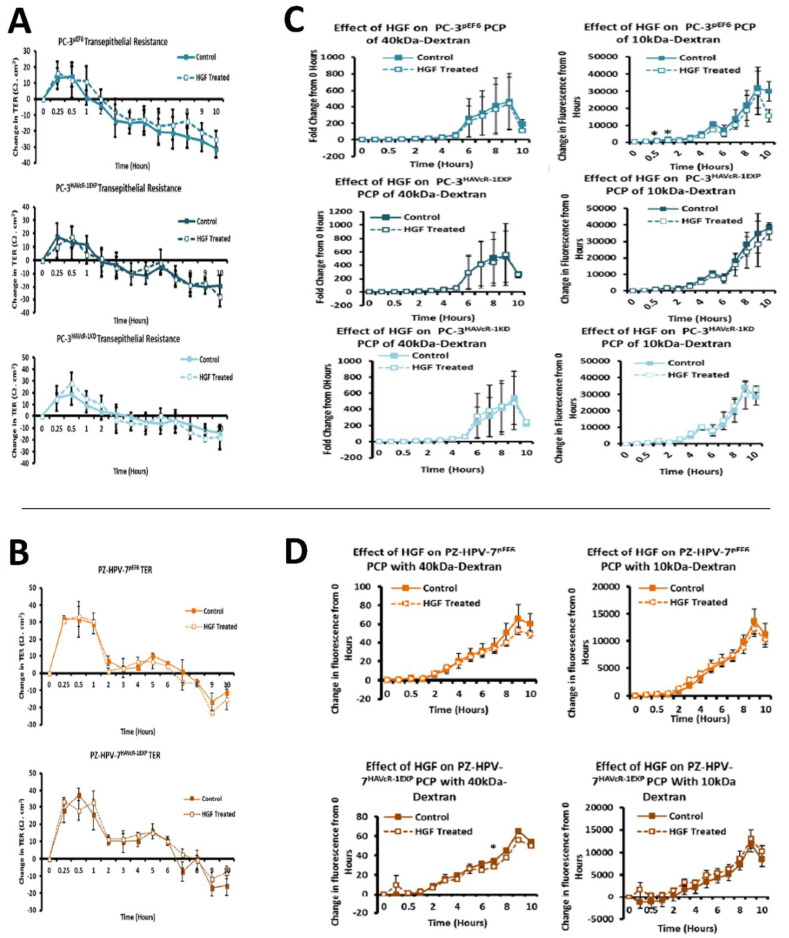
Comparison of HAVcR-1 expression modulation and treatment with HGF (40 ng/mL) on prostate cancer cell barrier function, TER and PCP. (**A**) Effect of HGF and HAVcR-1 on PC-3 transepithelial resistance (TER). Post incubation cells were treated with either of 40 ng/mL HGF or equal volumes of 0.1% BSS in PBS and resistance across the membrane was measured every hour for 10 h. Graphs show the means of three independent experiments as change relative to the resistance at 0 h for PC3pEF6, PC-3 HAVcR-1EXP and PC-3 HAVcR-1KD. Error bars show SEM. (**B**) Effect of HGF and HAVcR-1 on PZ-HPV-7 TER. Graphs show the means of three independent experiments as change relative to the resistance at 0 h for A PZ-HPV-7 pEF6 and B PZ-HPV-7 HAVcR-1EXP. Error bars show SEM. (**C**) Effect of HGF and HAVcR-1 on PC-3 paracellular permeability (PCP). Cells were treated with 40 ng/mL HGF or equal volumes of 0.1% BSS in PBS alongside 0.2 mg/mL of both TRITC–dextran (40 kDa) and FITC–dextran (10 kDa), and samples were taken every hour for 10 h. Graphs show the means of three independent experiments as fold change relative to fluorescence at 0 h of the 40 kDa FITC–dextan conjugate and the 10 kDa TRITC–dextan conjugate for PC-3 pEF6, PC-3 HAVcR-1EXP and PC-3 HAVcR-1KD. Error bars show SEM. (**D**) Effect of HGF and HAVcR-1 on PZ-HPV-7 PCP. Graphs show the means of three independent experiments as fold change relative to fluorescence at 0 h of the 40 kDa FITC–dextan conjugate and the 10 kDa TRITC–dextan conjugate of PZ-HPV-7 pEF6 and PZ-HPV-7 HAVcR-1EXP. Error bars show SEM. * represents *p* ≤ 0.05.

HGF had no significant effect on PC-3 pEF6 TER at any time point assessed, as shown in Figure 9A. The linear regression analysis showed no significant difference between the slopes of HGF-treated versus control (−3.20 ± 0.52 vs. −3.89 ± 0.53; *p* = 0.353). HAVcR-1 overexpression in PC-3 cells had no effect on this with no significant difference shown at any time point in the HGF-treated PC-3 HAVcR-1EXP in comparison to the control PC-3 HAVcR-1EXP, and the linear regression analysis showing no significant difference between slopes (−2.50 ± 0.50 vs. −3.47 ± 0.58; *p* = 0.214), as shown in Figure 9A. The HAVcR-1 knockdown in PC-3 cells also had no effect with no change in TER in the HGF-treated PC-3 HAVcR-1KD versus control PC-3 HAVcR-1KD at any other time point, and the linear regression analysis showed no significant difference between slopes (−3.37 ± 0.64 vs. −3.183 ± 0.62; *p* = 0.838), as shown in Figure 9A. HGF also had no significant effect on PZ-HPV-7 pEF6, Figure 9B (top), and the linear regression analysis showed no significant difference between slopes of the HGF-treated versus control (−3.47 ± 0.58 vs. −3.89 ± 0.53; *p* = 0.593). There was also no significant change in the HGF-treated PZ-HPV-7 HAVcR-1EXP in comparison to the control PZ-HPV-7 HAVcR-1EXP at any time point, and the linear regression analysis showed no significant difference between slopes (−3.20 ± 0.52 vs. −3.18 ± 0.62; *p* = 0.985), as shown in Figure 9B (bottom). It appeared that HGF had no effect on the barrier function whether or not HAVCR-1 was overexpressed or knocked-down.

When assessing changes in PCP, HGF exhibited no effect on the PCP of 40 kDa TRITC–dextran conjugate in PC-3 pEF6 versus control at any time point assessed, and no significant difference between slopes (35.56 ± 11.21 vs. 42.03 ± 12.24; *p* = 0.668), as shown in Figure 9C (left). The HGF treatment in PC-3 HAVcR-1EXP cells also had no effect on the PCP of 40 kDa TRITC–dextran conjugate with no significant difference in the HGF-treated PC-3 HAVcR-1EXP versus control PC-3 HAVcR-1EXP, and the linear regression analysis showed no significant difference between slopes (50.16 ± 14.72 vs. 51.62 ± 13.88; *p* = 0.942). Furthermore, the HGF treatment in PC-3 HAVcR-1KD cells also had no effect on the PCP of 40 kDa TRITC–dextran conjugate in the HGF-treated PC-3 HAVcR-1KD versus control PC-3 HAVcR-1KD, and the linear regression analysis showing no significant difference between slopes (46.46 ± 12.82 vs. 44.49 ± 11.47; *p* = 0.910), as shown in Figure 9C (Left). HGF significantly decreased the PCP of 10 kDa FITC–dextran conjugate in PC-3 pEF6 versus control at 0.5 h (508.05 ± 59.49 vs. 834.62 ± 58.01; *p* = 0.017) and at 1 h (1035.12 ± 90.63 vs. 1961.68 ± 131.33; *p* = 0.022), as shown in Figure 9C (right), though the linear regression analysis showed no significant difference (2205 ± 376.0 vs. 3027 ± 381.3; *p* = 0.129). The HGF treatment in PC-3 HAVcR-1EXP cells had no effect on the PCP of 10 kDa FITC–dextran conjugate, no significant difference was shown at any time point in the HGF-treated PC-3 HAVcR-1EXP versus control PC-3 HAVcR-1EXP and the linear regression analysis showed no significant difference between slopes (3194 ± 414.1 vs. 3666 ± 372.2; *p* = 0.399). There was no significant difference shown in the HGF treated in PC-3 HAVcR-1KD cells PCP of 10 kDa FITC–dextran conjugate, no significant difference shown at any time point in HGF-treated PC-3 HAVcR-1KD in comparison to control PC-3 HAVcR-1KD and the linear regression analysis showed no significant difference between slopes (3097 ± 292.7 vs. 2968 ± 293.3; *p* = 0.756), as shown in Figure 9C (right). We concluded that although HAVcR-1 expression was linked to effective changes in paracellular permeability, overall, HGF did not affect changes in PC-3 cells.

HGF had no effect on the PCP of 40 kDa TRITC–dextran conjugate in PZ-HPV-7 pEF6 in comparison to control at any time point assessed, although there was a significant decrease in the slope of HGF-treated PZ-HPV-7 pEF6 versus control PZ-HPV-7 pEF6 (5.40 ± 0.21 vs. 6.64 ± 0.56; *p* = 0.041), as shown in Figure 9D (left). The HGF treatment in PZ-HPV-7 HAVcR-1EXP cells did result in a decreased PCP of 40 kDa TRITC–dextran conjugate versus control at the 7 h time point (28.37 ± 1.17 vs. 34.58 ± 1.38; *p* = 0.028). The linear regression analysis showed a decrease between the slope of the HGF-treated PZ-HPV-7 HAVcR-1EXP and control PZ-HPV-7 HAVcR-1EXP (5.03 ± 0.32 vs. 6.11 ± 0.25; *p* = 0.010), as shown in Figure 9D (right). There was little effect with the HGF treatment on the PCP of 10 kDa FITC–dextran conjugate in PZ-HPV-7 pEF6 versus control, as shown in Figure 9D (right), and the linear regression analysis showed no significant difference between the slopes of HGF-treated comparison to control (1176 ± 67.07 vs. 1311 ± 88.86; *p* = 0.228). In contrast to PC-3 cells, HGF was able to affect differential changes in paracellular permeability in PZ-HPV-7 cells.

## 4. Discussion

Prostate cancer is a significant problem throughout the world, and due to the high-incidence rates can result in a large proportion of people burdened with the disease. Current diagnostic testing fails to meet the requirements for effective screening and lack an understanding of the progression of prostate cancer to metastatic disease. There is still a requirement for novel biomarkers to improve the diagnosis, monitoring and prognostic indicators to increase our understanding of disease progression, with the hopes of developing therapeutic targets for the treatment or prevention of metastatic prostate cancer. 

This current study initially examined serum HAVcR-1 ectodomain levels in prostate cancer, which showed decreased serum HAVcR-1 ectodomain levels in comparison to healthy control levels, and a significance decrease in serum HAVcR-1 ectodomain levels in the Gleason score of 6, 7, 8, 9 and 10 prostate cancers, in comparison to healthy control levels. This, therefore, presents HAVcR-1 as a potential diagnostic biomarker, which would be of particular interest where the current biomarker (PSA) is highly nonspecific [3]. Whether this is unique to prostate cancer has yet to be determined, and it may be possible that serum levels decrease in a variety of cancer types and, thus, HAVcR-1 may need to be used in conjunction with other biomarkers such as PSA in the instance of prostate cancer or followed by further testing to determine the cancer type. A more recent study has shown that HAVcR-1 was significantly upregulated at the mRNA and protein level in gastric cancer tissues compared to the adjacent normal tissues, and was an independent indicator of shorter overall survival, an important indicator that HAVcR-1 could be considered as a novel prognostic indicator in this cancer type [15]. It is also important to note that these are preliminary results and further testing is required prior to HAVcR-1 being used in this capacity, including greater sample numbers and trials to investigate whether serum HAVcR-1 levels can accurately diagnose prostate cancer as well as stage the disease. In clear cell renal cell carcinoma, there is a known link between the HAVcR-1 ectodomain shedding effect invasiveness and tumour malignancy, and it would, therefore, be interesting to investigate if a similar effect is seen in prostate cancer [16].

This study also aimed to assess the total HAVcR-1 expression in prostate cancer, which revealed a significant increase in HAVcR-1 protein expression in prostate cancer tissue samples in comparison to normal control samples. This result was unsurprising as it has previously been documented that there is increased HAVcR-1 staining in prostate cancer tissue samples [17]. Furthermore, using GEO datasets, HAVcR-1 overexpression in prostate cancer was also shown at the gene level. HAVcR-1 overexpression has previously been observed in breast cancer, ovarian cancer and renal cell carcinoma; therefore, providing further evidence that HAVcR-1 is not specific to a certain cancer type [16,18]. Interestingly, this increase in total HAVcR-1 protein expression was the opposite of the observed decreased serum HAVcR-1 ectodomain levels. Two possible explanations for this are that either the cleavage event that resulted in the release of the ectodomain was decreased in prostate cancer or there was a decreased entry of the HAVcR-1 ectodomain into the circulation in prostate cancer. A decreased cellular cleavage appears unlikely due to previously documented increased urinary HAVcR-1 levels with the occurrence of prostate cancer [13]. In regard to decreased entry into the circulation as HAVcR-1 is expressed in prostate glandular epithelial cells, it would be expected that, similarly to PSA, the disruption of the normal prostate architecture that occurs with prostate cancer progression would cause increased entry into the circulation [19]. A possible explanation as to why this was not the case is that the HAVcR-1 ectodomain is sequestered within the tumour. HAVcR-1 is expressed on the surfaces of CDK4+ T cells, CDK8+ T cells, natural killer (NK) cells, NKT cells, dendritic cells, B cells and mast cells [20,21]. HAVcR-1 is also a co-stimulatory molecule with ligand binding, resulting in the activation, proliferation and cytokine production of T cells and the activation of NKT cells [21,22,23] and ligands, including TIM-4 and phosphatidylserine (PS) [20,22,23]. Because HAVcR-1 can bind PS, it is possible that the released HAVcR-1 ectodomain was sequestered within the tumour, binding to TIM-4 and PS, preventing the activation of infiltrating immune cells [20]. If this was the case, the release of the HAVcR-1 ectodomain may contribute to the nonresponsiveness of many tumour-infiltrating immune cells and would be of interest for future study. HAVcR-1 is overexpressed in prostate cancer and, therefore, the staining of prostate biopsies could be used to aid in prostate cancer diagnosis; however, it would be of interest to investigate whether there is any correlation between the total HAVcR-1 expression and disease prognosis, as this would have more clinical benefit. 

PC-3, Du145 and LNCaP were assessed to model metastatic disease, CA-HPV-10 to model localised disease, and PZ-HPV-7 to model normal prostate epithelia. HAVcR-1 gene expression increased in PC-3, Du145, LNCaP and CA-HPV-10 cell lines in comparison to PZ-HPV-7, with this change being significant in PC-3 cells in comparison to LNCaP, CA-HPV-10 and PZ-HPV-7 cells. There was also a consistent increase in the total HAVcR-1 protein expression in PC-3, Du145, LNCaP and CA-HPV10 cells in comparison to PZ-HPV-7 cells. Therefore, a similar trend was seen in cell lines as in the clinical samples, that HAVcR-1 is overexpressed at the protein and gene level in prostate cancer. Moreover, there was no change in HAVcR-1 ectodomain levels found in cell media between cell lines; thus, conferring with the clinical data theory that the variation in serum HAVcR-1 ectodomain levels with the occurrence of prostate cancer is not due to a variation in the amount of HAVcR-1 cleavage. Cell line expression, therefore, agreed with the clinical data to a suitable degree that they could be used for further study into the effect of HAVcR-1 in prostate cancer.

We also set out to evaluate the effect of HAVcR-1 on prostate cancer cell behaviours that were imperative for metastasis to occur. Consistent with previous HAVcR-1 studies in colorectal cancer, HAVcR-1 had no significant effect on cell growth [10]. However, unlike the colorectal study, which showed that increased HAVcR-1 decreased invasion and adhesion, this study showed no significant change in either with HAVcR-1 overexpression or knockdown. HAVcR-1 overexpression in colorectal cells resulted in no change in cell migration; however, overexpression in PC-3 showed a potential decrease in wound healing and, therefore, proposes HAVcR-1 as a tumour suppressor [10].

In regard to TJ functions and the potential role that HAVcR-1 might have, PC3HAVcR-1EXP and PC3HAVcR-1KD cell models were utilised in a series of assays for the measurement of TJ integrity. Overall, these assays showed no change in resistance with PC3HAVcR-1EXP or PC3HAVcR-1KD, suggesting that HAVcR-1 has no effect on TJ integrity and is inconsistent with the hypothesis that the increased HAVcR-1 expression seen in prostate cancer is important for metastasis to occur via the decreased integrity of TJs. The effect of HAVcR-1 on paracellular permeability was also assessed due to TJs being the primary determinant of epithelial permeability, with Claudin expression patterns in particular being responsible for pore selectivity [24]. However, HAVcR-1 appeared to have no effect on PC-3 permeability and, thus, further suggesting that HAVcR-1 expression has no bearing on the integrity of TJ within PC-3 cells or on the composition of TJs within PC-3 cells. The compositional stability of PC-3 TJs with manipulated HAVcR-1 expression was further validated with the gene expression of all TJ proteins investigated remaining constant. Preliminary investigations into TJ protein expression showed minute changes in the expression and localisation of occludin and ZO-1. Decreased occludin staining in PC-3 HAVcR-1EXP and increased staining in PC-3 HAVcR-1KD suggests that the overexpression of HAVcR-1 would decrease TJ integrity and that targeting HAVcR-1 could, therefore, be a potential therapeutic target for prostate cancer. However, contradictory to this, there was also an increase in occludin membranous staining with HAVcR-1 overexpression, suggesting an increase in TJs. Furthermore, in PC-3 cells that overexpressed HAVcR-1, there was a decreased nuclear staining of ZO-1. ZO-1 contains both NLS and NES; thus, can shuttle between TJs and the cell nucleus [25]. Nuclear levels are generally associated with a decreased TJ integrity being found in proliferating low confluent cells [26]. Therefore, low ZO-1 nuclear staining further suggests an increased junctional stability with HAVcR-1 overexpression. There was, however, a decrease in the barrier resistance with HAVcR-1 overexpression and knockdown during cell adhesion and spreading, indicating a decrease in the cell–cell junction integrity. TER results suggested that the junctional integrity remained constant regardless of HAVcR-1 expression; it is a possibility that HAVcR-1 levels affect the initiation of junction assembly, but do not affect the integrity of junctions once formed. The cell adhesion assay as well as the ECIS initial attachment experiment showed no change in cell adhesion with a manipulated HAVcR-1 expression. However, there was an increased constraint under cells with decreased HAVcR-1 expression, suggesting a decreased focal adhesion. To validate the changes to focal adhesion, further analyses are required, such as conducting a dynamic culture cell adhesion assay [27]. There has been some research to indicate that HGF is important in HAVcR-1 activation and, therefore, it would be interesting to investigate the effect of a combination of HGF and HAVcR-1 on cell behaviours and TJ integrity [14]

The Kinex™ KAM880 protein array analysis indicated 64 proteins as being statistically altered, either in expression or phosphorylation. Although there were numerous proteins that would be of interest for further study, β-catenin was chosen. β-catenin Y333 showed a significantly increased phosphorylation with HAVcR-1 overexpression. As well as β-catenin being an important structure component of AJs, it also has a role in the de-differentiation process [28]. β-catenin has been shown to play a role in the tumorigenesis of numerous cancers, with dysregulation being associated with prostate cancer progression; however, studies have generally focused on the Wnt/wingless cascade and activation mutations [29]. However, the phosphorylation of β-catenin at residue Y333 was due to EGFR signalling; thus, identifying a novel area of interest in the study of prostate cancer research [30]. HAVcR-1 had no direct effect on the total gene or protein expression of β-catenin in PZ-HPV-7 cells, although HAVcR-1 overexpression altered the subcellular localisation of β-catenin, which is an important indicator of signalling [29]. We found an increase in the β-catenin nuclear staining, and since nuclear staining is indicative of β-catenin activation, this, therefore, supports the Kinex™ KAM880 protein array data and the theory that HAVcR-1 is involved in β-catenin signalling [31]. Interestingly, the nuclear accumulation of β-catenin has been associated with poorly differentiated and highly proliferative tumours, with an increased vascular invasion [29,30]. Since invasion is a hallmark of malignancy and a prerequisite for cancer metastasis, this proposes HAVcR-1 as a potential anti-metastatic target [32]. The Kinex™ KAM880 protein array also revealed an increase in the α-catenin S641 phosphorylation, again, as a result of EGFR activation and, subsequently, resulted in the dissociation of β-catenin from the membrane and its nuclear translocation [33]. The gene and protein analysis of α-catenin expression showed that HAVcR-1 had no effect on the total expression levels, although immunofluorescence showed a discontinuous staining of α-catenin at the cell membrane; thus, suggesting a breakdown of AJs, which was further shown by the discontinuous membrane staining of E-cadherin with HAVcR-1 overexpression. These results, therefore, support the Kinex™ KAM880 protein array and the theory that HAVcR-1 could lead to the phosphorylation of α-catenin, which, subsequently, results in the disassociation of β-catenin from AJs and nuclear accumulation. 

The junctional integrity was also shown to decrease with HAVcR-1 overexpression, with a decreased barrier resistance during wound healing and an increased PCP. Thus, it is plausible that HAVcR-1 results in a decreased junctional stability via the activation and, thus, the dissociation of β-catenin from AJs. Once accumulated in the nucleus, β-catenin binds PKM2 and this complex can be recruited to the CCND1 promoter leading to targeted gene transcription, including cyclin D1 [30,34]. HAVcR-1 had no direct effect of PKM2 gene expression; however, this was unsurprising due to the localisation of PKM2 being important in β-catenin signalling. The cyclin-D1 gene expression remained constant with HAVcR-1 overexpression; however, the protein expression significantly increased. It would be expected that activated β-catenin signalling would increase cyclin D1 transcription; therefore, increasing the cyclin D1 gene expression and protein expression [35]. Cyclin-D1 is a cell cycle control protein and has been linked to the development and progression of cancer. Cyclin D1 is a regulator of the progression of cells into the proliferation stage of the cell cycle, in LNCaP cells cyclin D1 overexpression enhancing S-phase entry, increasing colony formation and the tumour growth rate [35,36,37]. EMT is a multi-step process involving the decreased integrity of junctional complexes [38]. As previously discussed, immunofluorescence showed as decreased integrity of AJs with E-cadherin, α-catenin and β-catenin membrane staining.

The HGF treatment has been shown to increase the cell growth of KGN and HO8910 ovarian cancer cell lines and the PC-3 cell line [39,40,41]. HAVcR-1 overexpression and knockdown had no effect on this with no change in the cell growth with the HGF treatment. HGF has also been shown to influence cell migration with the HGF treatment increasing the cell migration of HO8910 ovarian cells line, the MKN1, MKN7 and MKN28 gastric cell lines and the PC-3 cell line [39,40,41,42]. However, there was no change in the cell migration of PC-3 pEF6 or PZ-HPV-7 pEF6 cells. Although HAVcR-1 overexpression had no effect on this with no change in PC-3 cell migration with the HGF treatment, the HAVcR-1 knockdown increased the PC-3 cell migration when treated with HGF. This, therefore, suggests that HAVcR-1 inhibited the ability of HGF to influence cell migration. However, HAVcR-1 overexpression in PZ-HPV-7 cells resulted in an increase in cell migration with the HGF treatment, which suggests that HAVcR-1 is required for HGF to influence cell migration. It is, therefore, possible that the control of HAVcR-1 levels is essential with only a small range in expression allowing HGF signalling. HGF had no effect on the PC-3 pEF6 cell adhesion and an inhibitory effect on PZ-HPV-7 pEF6 cell adhesion; however, this contrasted a previous report, whereby HGF had been shown to increase PC-3 cell adhesion [39]. HAVcR-1 knockdown, however, resulted in an increased PC-3 adhesion with the HGF treatment, which further suggests that HAVcR-1 has an inhibitory effect on HGF signalling in PC-3 cells due to HAVcR-1 overexpression also resulting in a decreased cell adhesion of PZ-HPV-7 cells, causing the HGF signalling in PC-3 and PZ-HPV-7 to be different. The effect of the HGF treatment on cell invasion has previously had conflicting results, with some showing the treatment resulting in an increased cell line invasion (HO8910 and PC-3) and others showing that the cell line (PC-3 and PZ-HPV-7) invasion was unaffected by the treatment [39,41,43]. Similarly, to the latter, the HGF treatment had no effect on PC-3 pEF6or PZ-HPV-7 pEF6 cell invasion. HAVcR-1 overexpression and knockdown in PC-3 cells as well as HAVcR-1 overexpression in PZ-HPV-7 had no effect on this, with no difference in invasion with the HGF treatment. These results, therefore, suggest that change to cell invasion in prostate cancer cells is independent of HGF signalling. Previous studies had demonstrated a decreased TER and decreased TJ protein expression and membrane localisation and, therefore, a decreased junctional integrity with the HGF treatment [44,45]. There was no effect with the HGF treatment when HAVcR-1 was overexpressed or knocked-down in PC-3 cells or when overexpressed in PZ-HPV-7 cells. This was contradictory to the literature, whereby the knockdown of HAVcR-1 resulted in HECV cells being resistant to an HGF-induced decreased TER [14]. As HECV cells are endothelial and PC-3 and PZ-HPV-7 are epithelial derived, the regulation mechanisms of junctional integrity may differ between endothelial and epithelial cells. This is further supported by the potential initial decrease in the PCP of 10 kDa FITC–dextran conjugate of PC-3 pEF6 with the HGF treatment, which would suggest an increase.

## 5. Conclusions

This study showed that serum levels of the HAVcR-1 ectodomain varied in prostate cancer and, therefore, identified a novel area of study in prostate cancer diagnosis and monitoring. Future studies could assess the potential benefits of using serum levels in blood tests in a clinical setting, as well as evaluating the variations in signalling pathways, resulting in the release of HAVcR-1 from prostate cancer cells. HAVcR-1 could be developed as a biomarker, particularly using ectodomain shedding in the future. This work also demonstrated that HAVcR-1 has the capacity to alter cell behaviour to promote pro-cancer and pro-metastatic phenotypes. This was illustrated by the changes in HGF-mediated signalling made possible via the catenin complex and affecting changes in the barrier function and, hence, increases in aggressive phenotypes of human prostate cancer cell lines. These potential signalling pathways and further studies on inhibitors to HAVcR-1-induced cell behavioural changes and the signalling pathways activity are required to potentiate the HAVcR-1 inhibition as a treatment of prostate cancer and/or prevention of metastatic disease.
